# Physical Vapor Deposition in Solid‐State Battery Development: From Materials to Devices

**DOI:** 10.1002/advs.202002044

**Published:** 2021-03-19

**Authors:** Sandra Lobe, Alexander Bauer, Sven Uhlenbruck, Dina Fattakhova‐Rohlfing

**Affiliations:** ^1^ Forschungszentrum Jülich GmbH Institute of Energy and Climate Research: Materials Synthesis and Processing (IEK‐1) Wilhelm‐Johnen‐Straße Jülich 52425 Germany; ^2^ Helmholtz Institute Münster: Ionics in Energy Storage (IEK‐12) Jülich 52425 Germany; ^3^ Faculty of Engineering and Center for Nanointegration Duisburg‐Essen (CENIDE) Universität Duisburg‐Essen (UDE) Lotharstraße 1 Duisburg 47057 Germany

**Keywords:** interface, physical vapor deposition, solid‐state battery, thin film electrode, thin film electrolyte

## Abstract

This review discusses the contribution of physical vapor deposition (PVD) processes to the development of electrochemical energy storage systems with emphasis on solid‐state batteries. A brief overview of different PVD technologies and details highlighting the utility of PVD for the fabrication and characterization of individual battery materials are provided. In this context, the key methods that have been developed for the fabrication of solid electrolytes and active electrode materials with well‐defined properties are described, and demonstrations of how these techniques facilitate the in‐depth understanding of fundamental material properties and interfacial phenomena as well as the development of new materials are provided. Beyond the discussion of single components and interfaces, the progress on the device scale is also presented. State‐of‐the‐art solid‐state batteries, both academic and commercial types, are assessed in view of energy and power density as well as long‐term stability. Finally, recent efforts to improve the power and energy density through the development of 3D‐structured cells and the investigation of bulk cells are discussed.

## Graphical abstract

The review discusses how the development of electrochemical energy storage systems and particularly solid‐state batteries can benefit from physical vapor deposition processes, from the basic understanding of the structure and properties of individual materials and their interfaces to the processing and fabrication of complete batteries.

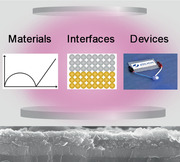

## 1 Introduction

Advanced electrochemical energy storage is internationally considered as one of the disruptive technologies of the future.^[^ ^1^ ^]^ The introduction of lithium ion battery technology boosted the available energy density of battery packs, and tremendous progress regarding the performance of lithium ion batteries has been achieved in the past decades to enable even very demanding applications for energy storage, such as, electric vehicles. Still, the demand for even higher storage capacity, faster charging rates, prolonged operational life, and more stringent safety standards require the development of more powerful electrochemical energy storage concepts. These challenges perpetuate vigorous research efforts on all levels of battery development, which remains one of the focal points of energy research.

In this review it is shown how the development of electrochemical energy storage systems can benefit from physical vapor deposition (PVD) processes, from the basic understanding of the structure and properties of individual materials and their interfaces to the processing and fabrication of complete batteries. PVD is a process in which material is deposited from the gas phase. Its key advantage is the creation of dense solid layers with a tunable thickness, adjustable and controlled composition, crystallinity and crystal orientation. Further, the risk of contamination is minimal due to the absence of organic reactants, and there is the option to sequentially deposit several materials to form well‐defined multilayer systems. One of the unique advantages of PVD is the operation at significantly lower processing temperatures relative to the densification of materials by conventional heat treatment due to the higher energy of the species in the gas phase. This is particularly important (or even essential) when two materials to be processed as adjacent layers are prone to mutual interdiffusion or detrimental chemical reactions.

PVD techniques are highly useful for addressing various aspects of development within a broad spectrum of battery concepts.^[^ ^2^ ^]^ However, in this review, the authors focus mainly on ceramic solid‐state battery research and development for which PVD methods have shown to unfold their full potential. Therefore, the discussion includes lithium metal and Li‐ion batteries, but it does not touch upon Li‐sulfur or Li‐oxygen systems.

Following a brief overview of different PVD technologies, the application of PVD for processing of individual battery materials is discussed in detail. We summarize the key processes that have been developed for the fabrication of solid electrolytes as well as active electrode materials with different crystalline structures and analyze how the processing conditions affect the resulting material characteristics. PVD techniques can be utilized to expand the fundamental understanding of battery materials (such as, e.g., the influence of the crystalline structure, crystal orientation and defects on the electronic and ionic conductivity, electrochemical performance, and underlying material transformations). Further, the application of PVD for the development of novel materials via the generation of material libraries that enable high‐throughput material screening is illustrated. The fabrication of thin film battery components, such as thin separator layers and various coatings for different battery designs, is also discussed.

With respect to the complex interfacial phenomena encountered in solid‐state batteries we present work that demonstrates how PVD facilitates both understanding and optimization of interfaces between different battery materials. Due to the possibility of sequentially depositing planar layers at low temperatures, PVD is ideally suited for fabricating model systems to investigate interfaces between solid materials. Model systems obtained via PVD techniques enable the deconvolution of interfacial phenomena that occur during processing and operation. Aside from discussing fundamental studies of interfacial properties, we describe the application of PVD for the production of thin functional layers, which improve the properties of the interfaces. More specifically, protective coatings that prevent chemical or electrochemical reactions between battery materials during processing or operation are described as well as adhesive coatings, which improve the contact between the materials and thus minimize the interfacial resistance.

Due to its ability to control the properties of individual materials and their interfaces, and by allowing the fabrication of multi‐layer structures, PVD technology is an excellent tool for the development of thin film batteries, which are also described in this review. Properties of state‐of‐the‐art commercial thin film batteries are briefly discussed as well as ongoing efforts to further improve the battery performance and also enable cell designs for specialty applications. Finally, to provide a comprehensive overview of processing issues with respect to the development of ceramic batteries, a brief summary regarding the progress of bulk battery cell development to date is provided.

## 2 Physical Vapor Deposition

### 2.1 Methods

PVD refers to a variety of vacuum techniques used to deposit thin films by the transport of material from a condensed matter source via the gas phase to another surface that shall be coated. The physical properties of the material generally do not change. In contrast to conventional ceramic processing, where materials have to be heat‐treated or densified at high temperatures (above roughly 50–75% of the melting temperature of the material^[^ ^3^ ^]^), PVD can provide dense and crystalline films at significantly lower temperatures. This is especially advantageous in setups where element interdiffusion between adjacent layers is detrimental and has to be avoided, for example, for model systems that are applied to study interfacial phenomena. As an example, a sintering temperature of at least 1400 °C is needed for the densification of screen‐printed yttria‐stabilized zirconia,^[^ ^4^ ^]^ whereas comparably dense layers are obtained at 800 °C by PVD.^[^ ^5^ ^]^

PVD techniques generally involve processes featuring a ballistic transport of material. Ballistic transport means a transfer of material from the source to the sample without any or only very few collisions with other atoms, ions or molecules. The species transferred from the condensed phase to the gas phase obtain energy from the material source, which is the basis for the creation of dense layers compared to conventional heat treatment during ceramic processing. This energy gain has to be preserved until the gas species hit the sample surface. Hence, a ballistic transport requires a mean free path of roughly a couple of centimeters to a couple of decimeters. This implies pressure ranges of less than about 1 to 10 Pa.^[^ ^6^ ^]^

**Figure 1:**
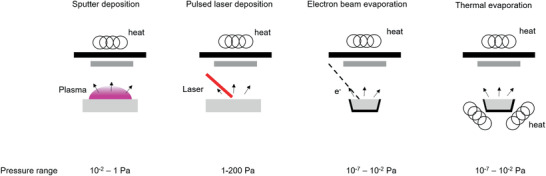
Schematic illustration of different PVD techniques.

The deposition rates in PVD‐processes are highly dependent on the processing conditions and typically range between 1 and 100 nm s^−1^. Accordingly, this leads to layer thicknesses from a few nanometers to hundreds micrometers with process times varying from fractions of seconds to hours. For solid‐state battery research, the most important PVD processes are sputtering, pulsed laser deposition (PLD) and evaporation (thermal and electron beam) techniques. Simplified schemes of the single processes are shown in **Figure** **1**. Sputter deposition and evaporation processes are already well‐established in industrial processes (thermal insulation on architectural glass, wear‐resistant tools) due to their ability to coat large areas, including roll‐to‐roll processes, whereas PLD was limited to small substrate sizes for many years. In the last decade, the available areas for coating increased up to 300 mm diameter. This improvement was realized by beam widening and shaping, with orders of magnitude higher output powers compared to typical PLD laser systems for research.^[^ ^7^ ^]^

Vacuum and energy uptake during the phase transition from the condensed phase to the gas phase are important aspects and are discussed in more detail in the energy considerations in the following sections in which the fundamental characteristics of widely applied PVD techniques are described.

#### 2.1.1 Sputter Deposition

Sputtering is the acceleration of gas ions on the surface of the material source where the material is spalled off/atomized. A high electric field is used to create a plasma. The setup for sputtering features an electrical current flow. A direct current (d.c.) method can be utilized for reasonably conductive material sources, whereas radio frequency (r.f.) currents are required in the case of electrical insulators. Plasmas are comparatively difficult to describe, as they can exist in thermal non‐equilibrium conditions. The electric field properties and intricacies of the sputter setup influence the generation of the plasma, especially the temperature, which in turn directly affects the velocity and energy distribution and also the collision cross sections of the species in the gas phase.^[^ ^8^ ^]^ In general, the energy of the particles in the vapor phase during sputtering is higher compared to conventional evaporation due to the energy stemming from the high electric field,^[^ ^9^ ^]^ so that deposition can be carried out at higher background pressures.

So‐called reactive sputtering processes represent a special case. Here, a gas with a slightly higher pressure than what would be common for conventional PVD is supplied, and the species sputtered from the target react with the gas molecules, thus forming new compositions. Therefore, reactive sputtering differs in two major aspects from the sputtering process described above: i) The target, gas, and layer composition are not identical, in contrast to conventional PVD; ii) the sputtered species collide with the gas molecules, which is intentional, in order to interact/react with them. Therefore, it is necessary to change the process, for example, to increase slightly the gas pressure, which allows more collisions. Thus, the gas transport behavior is shifted to a transition regime between a purely ballistic regime and a purely diffuse regime.

#### 2.1.2 Evaporation Techniques

The most well‐known process for transferring material from a condensed phase to a gas phase is evaporation, resulting in a gas phase above the condensed phase. In an ideal case the gaseous species are in at least a local thermal equilibrium, and their velocities can be described by a Maxwell–Boltzmann distribution, although deviations from classical statistical mechanics were also reported.^[^ ^10^ ^]^ Temperature is a significant parameter for the velocity distribution and thus for the energy distribution of the particles hitting the sample surface during deposition.

The most common method for evaporation is Joule heating, by which electrical energy is converted into heat by a resistor. This setup is limited to temperatures that are lower than the melting points of the crucibles and heating wires. By contrast, electron beam evaporation is also capable of evaporating refractory materials like highly temperature‐resistant ceramics. A focused electron beam is wobbled along the source material surface for heating. Further, effusion or Knudsen cells are specifically designed evaporation devices through which the vapor beam may be generated by Joule heat, but a directed beam is created by effusion.

While evaporation is one of the simplest methods to transfer materials in a gaseous phase, it is not universally applicable to the deposition of multicomponent compounds. Major problems arise if the compound to be evaporated melts incongruently, which may lead to variations in the compositions of the molten phase and the gas phase.^[^ ^11^ ^]^

#### 2.1.3 Pulsed Laser Deposition

PLD evaporates and ionizes the material through one or a series of high energy laser pulses. The problem of compositional changes often encountered with evaporation processes practically does not arise. Therefore, PLD is favorable for the stoichiometric deposition of materials. As the laser light is focused on a small spot, only small areas of a sample can be coated in a homogeneous manner. The area‐specific power density of the laser pulse can be as high as 10^15^ W m^−2^, assuming pulses of 10 J in10 ns on an area of 1 mm^2^. This can be compared with the energy dissipation of 10^20^ W of a nuclear bomb,^[^ ^12^ ^]^ which also highlights the difficulty of scaling up PLD to coat large areas. The fact that PLD does not only evaporate the material, but also ionize the vapor, illustrates the high vapor particle energy during processing,^[^ ^13^ ^]^ which enables deposition at higher pressures, similar to sputtering processes. Therefore, reactive gases, which play an important role in the deposition of oxides, can be introduced into the deposition chamber.

### 2.2 Thin Film Growth

**Figure 2:**
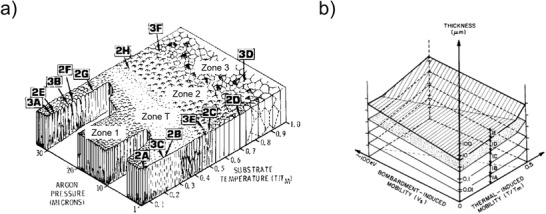
Zone growth models from a) Thornton, Reproduced with permission.^[^ ^14^ ^]^ AIP Publishing. b) Messier et al. Reproduced with permission.^[^ ^15^ ^]^ Copyright 1984, American Vacuum Society.

The morphology developing during thin film growth depends on several factors, which are related to the mobility of the atoms on the substrate surface. Zone growth models (ZGM) were developed to describe the most important parameters. The most widely known ZGM is most likely the model developed for thick sputtered films by Thornton, which takes into account the process pressure and the ratio between the deposition temperature *T* and the melting temperature of the sputtered material *T*_m_ (*T*/*T*_m_) as the factors defining the film growth (**Figure** **2**a).^[^ ^14^ ^]^ Four zones are described in the model:

‐ Zone 1 (*T*/*T*_m_ < 0.1): Low adatom mobility, small grains separated by voids.
‐ Transition zone (0.1 < *T*/*T*_m_ < 0.3): Dense arrays of fibrous grains.
‐ Zone 2 (0.3–0.5 < *T*/*T*_m_ < 0.75): Columnar grains, grain boundary migration, and recrystallization possible.
‐ Zone 3 (0.75 < *T*/*T*_m_): Flat grain tops with grooved grain boundaries.

Messier et al. further developed the model for *T*/*T*_m_ < 0.3–0.5 including films with a thickness below 1 µm.^[^ ^15^ ^]^ The five zones 1A, 1B, 1C, 1D, and 1E correspond to the growth of clusters with column sizes of 1–3, 5–20, 20–40, 50–200, and 200–400 nm, respectively (Figure 2b).^[^ ^15^ ^]^ The nanostructured/amorphous nature of thin films with high‐melting compositions can be explained with this model. In the model developed by Messier et al., the bombardment‐induced mobility, which can be practically implemented by ion‐beam assistance or bias sputtering, was also mentioned as one factor that can improve the crystallization of thin films.^[^ ^15^ ^]^

The deposition temperature and the energy of the impinging particles further influence the interface formation between the substrate and the thin film.^[^ ^16^ ^]^ The smaller these factors are, the smaller the resulting interface thickness becomes and vice versa. In order to deposit epitaxial thin films, the interface formation between the substrate and the thin film should be limited to a minimum. However, substrate heating is necessary to enable a sufficient mobility of the deposited atoms and thus the growth of a 2D film. Epitaxial growth can be realized more effectively by PLD or evaporation.

### 2.3 Material Development Facilitated by Physical Vapor Deposition

PVD techniques provide unique possibilities for high through‐put screening of novel materials and thus the generation of material libraries, which is one key task in current battery research. Films with tunable thickness, multielement composition, and composition gradients can be deposited in a controlled way. The thin film geometry is perfectly suitable for rapid screening by a wide variety of characterization techniques. Recent developments in automatization of analytical processes^[^ ^17^ ^]^ and data processing in combination with well‐established technologies for thin film patterning, such as lithography, enable compiling large databases, which can be applied for machine learning processes. In addition to the development of battery components, which is discussed in more detail in the next chapter, the application of thin film libraries is well established for material development for photoelectrochemical water splitting,^[^ ^18^ ^]^ shape memory alloys,^[^ ^19^ ^]^ transparent conductive oxides,^[^ ^20^ ^]^ and several other technologies. For further reading on this subject a review by Kafizas and Parkin is recommended.^[^ ^21^ ^]^

**Figure 3:**
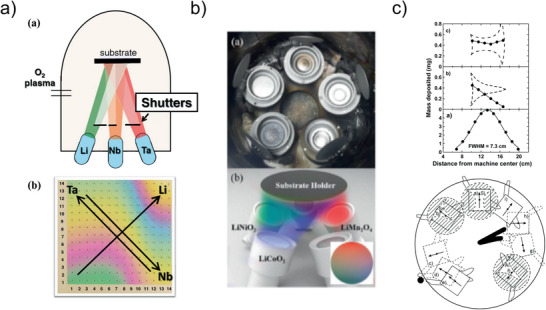
Different technical setups for the generation of material libraries by PVD. a) Setup for deposition of interlayers by using Knudsen‐effusion cells and oxygen plasma (top) and the generated sample from Li—Ta—Nb (bottom). Reproduced with permission.^[^ ^22^ ^]^ Copyright 2015, ECS. b) Setup for sputter deposition of up to five different materials at once, equipped for LiNiO_2_‐LiCoO_2_‐LiMnO_2_ gradients. Reproduced with permission.^[^ ^23^ ^]^ Copyright 2016, Elsevier. c) Effect of shadow masks to generate gradients(top) in a setup for up to 3 elements (Si—Sn—Al) (bottom). Reproduced with permission.^[^ ^24^ ^]^ Copyright 2002, American Chemical Society.

Typical techniques for the deposition of material libraries are shown in **Figure** **3**. The use of different material sources with different tilt angles/orientations will lead to a gradient due to the typical cosine distribution of the film thickness in PVD, provided that the substrate is not rotated. The slope of the gradient depends on the tilt angle of the sources. In Figure 3 examples for realizing such a gradient with effusion cells (Figure 3a) as well as in sputtering processes (Figure 3b) are shown. Intermixing of different materials by fast rotation of the substrate over different sputtering targets, as presented in Figure 3c, is also a possibility. In this case, the amount of material deposited from each target is controlled by using different shadow masks, where each mask causes a characteristic concentration curve.

## 3 Deposition and Properties of Thin Film Battery Components

The deposition of thin films with defined composition, crystallinity, orientation, and thickness is the key to a deeper understanding of material properties. However, the processing parameters have to be carefully adjusted to obtain appropriate samples. As already explained in Section 2.2, the degree of crystallization can be controlled very precisely by PVD, which enables the investigation of materials in their crystalline or amorphous state. However, the crystallization, which is a crucial step for a wide variety of battery materials, is carried out via heating, either in situ or in a post‐annealing step, which means that detrimental diffusion/reactions into/with the substrate material can occur. Further, if the substrate and the deposited layer are not compatible due to different coefficients of thermal expansion (CTE), bond character and/or lattice parameters, the risk of defects is increased with increasing treatment temperature. Therefore, a decrease of the deposition temperature, for example, by application of a bias voltage during a sputter process, is always desirable in thin film research.

The thin films deposited by PVD processes are free of organic additives and solvents and have a defined surface area, which allows relatively straightforward measurements of fundamental electrical and electrochemical properties. Further, PVD methods are suitable for the fabrication of films grown epitaxially or with preferred orientation, enabling the determination of material properties along a defined crystallographic axis. In addition, the analysis of chemical information, for example, by secondary ion mass spectrometry (SIMS) and X‐ray photoelectron spectroscopy (XPS), for thin film geometries is facilitated considerably.

Material libraries generated with PVD processes can be applied for high‐throughput material screening, which can accelerate the development of new compositions with enhanced electrochemical properties.

### 3.1 Deposition and Properties of Thin Film Electrolytes

The electrolyte plays a fundamental role in all types of batteries. In addition to ideally exhibiting a high Li‐ion conductivity and a low electronic conductivity it also should not form detrimental interfaces (e.g., by reaction) with electrode materials. The electrolyte does not take part in the electrochemical reaction, and therefore its mass should be reduced as much as possible to maximize the energy density of the cells. This can be realized by deposition of thin film electrolytes. Processing of thin films by PVD methods for the most common glass/ceramic electrolytes is discussed in this section.

#### 3.1.1 Li_3_PO_4‐_ *_x_*N*_x_*

The amorphous, partially nitrated phosphate Li_3_PO_4‐_ *_x_*N*_x_* (LiPON) was first described as a good lithium ion conductor by Bates et al. in 1996.^[^ ^25^ ^]^ In general, LiPON is deposited by an r.f. sputter deposition process from a Li_3_PO_4_ target by using nitrogen as a reactive sputter gas without substrate heating. PVD is considered to be the only technique to obtain LiPON layers with sufficient conductivity and satisfactory performance.

**Figure 4:**
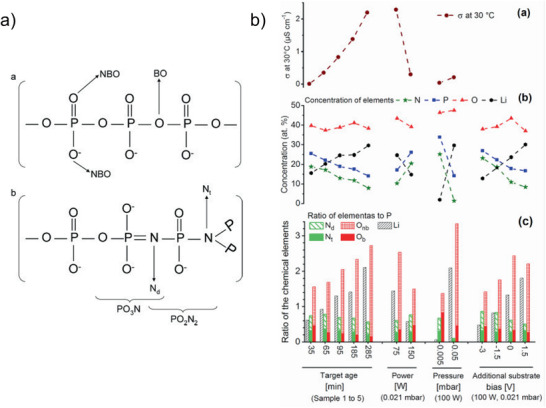
a) Structural features of LiPON, NBO = non‐bridging oxygen, BO = bridging oxygen, N_d_ = di‐coordinated nitrogen, N_t_ = tri‐coordinated nitrogen. Reproduced with permission.^[^ ^37^ ^]^ Copyright 2012, Elsevier. b) Correlation of various chemical and structural features with Li‐ion conductivity in LiPON thin films. Reproduced with permission.^[^ ^35^ ^]^ Copyright 2018, Elsevier.

The reported key properties of LiPON are an ionic conductivity of about 2 × 10^−6^ S cm^−1^ and an activation energy of about 0.55 eV.^[^ ^25^, ^26^ ^]^ Amorphous Li_3_PO_4_ thin films showed a conductivity of 6.3 × 10^−8^ S cm^−1^ and an activation energy of 0.67 eV.^[^ ^26^ ^]^ The highest conductivities reported for LiPON to date are 9.4 × 10^−6[^ ^27^ ^]^ and 9.78 × 10^−6^ S cm^−1^.^[^ ^28^ ^]^ On the other hand, the conductivities for crystalline Li_3_PO_4_ and LiPON are 4.2 × 10^−18^ and 1.4 × 10^−13^ S cm^−1^, respectively.^[^ ^29^ ^]^ In order to keep the thin films in the highly conductive amorphous state, substrate heating is not applied during LiPON deposition. The low deposition temperatures are beneficial for battery fabrication, as they prevent severe element interdiffusion, which is typically observed at high processing temperatures. Extensive work was done to deposit LiPON thin films with different sputter parameters, such as sputter power,^[^ ^30^, ^31^ ^]^ pressure,^[^ ^30^, ^32^ ^]^ nitrogen flow rate,^[^ ^33^ ^]^ bias voltage, and substrate temperature.^[^ ^34^ ^]^ The objective was to elucidate the influence of the chemical structure on the ionic conductivity. Different chemical features, such as the N/P, Li/P, and Li/O ratio, as well as structural features, such as the ratio between triply coordinated nitrogen (N_t_) and doubly coordinated nitrogen (N_d_), the amount of bridging oxygen atoms (**Figure** **4**a), etc., were discussed as the potential cause of high conductivity. However, although all chemical and structural features show trends when investigated as a single parameter, combined studies showed that the conductivity cannot be correlated with one single structural feature.^[^ ^28^, ^35^ ^]^ Hamedi Jouybari et al. concluded from their study that it is more likely that sufficient amounts of nitrogen and lithium incorporated in the structure and a low amount of bridging oxygen increase the conductivity (Figure 4b).^[^ ^35^ ^]^ These results were confirmed by theoretical and experimental work by Lacivita et al. on the structural features of LiPON. Their studies do not support the existence of N_t_ in the structure.^[^ ^36^ ^]^ Their work suggests the existence of apical N (N_a_) as the main structural feature that enables high ionic conductivity. The calculation fits neutron and infrared‐spectroscopy data very well, and a comparison of their results with conductivity data from the literature shows good agreement. However, this detailed study did not provide a complete explanation of the experimentally observed XPS spectra, which were mainly used to determine the structural features, and also did not explain some of the experimentally determined conductivity values, for example, the high conductivity of 9.78 × 10^−6^ S cm^−1^ published by Mani et al.^[^ ^28^ ^]^

While LiPON thin film fabrication employs a sintered Li_3_PO_4_ target in most studies, a few reports discussed the deposition from powder targets. Nimisha et al. and Suzuki et al. obtained LiPON thin films with conductivities of 1.1 × 10^−6[^ ^38^ ^]^ and 3.1 × 10^−6^ S cm^−1^,^[^ ^39^ ^]^ respectively, which is similar to values commonly published in the literature. On the other hand, a significant increase in the ionic conductivity was realized by adding Li_2_O to the powder target, so that conductivities of up to 6.4 × 10^−6^ S cm^−1^ could be achieved. However, the Li_2_O rich film showed more severe degradation after exposure to air, as pronounced particle growth, cracks and exfoliation were observed by scanning electron microscopy (SEM) analysis after 20 days.^[^ ^40^ ^]^

LiPON films were also grown by PLD from a Li_3_PO_4_ target in a flowing N_2_ atmosphere. A high pressure of about 267 Pa and a high laser fluence in the range of 15–20 J cm^−2^ were necessary to incorporate a sufficient amount of nitrogen into the glassy state, so that conductivities of up to 1.67 × 10^−6^ S cm^−1^ could be obtained.^[^ ^41^ ^]^ These observations are complementary with the low conductivities obtained from studies with lower nitrogen pressure and laser fluence.^[^ ^42^, ^43^ ^]^ Nitrogen‐free Li_3_PO_4_ deposited by PLD yielded conductivities of up to 4 × 10^−7^ S cm^−1^.^[^ ^42^ ^]^

Electron beam evaporation processes supported by a nitrogen plasma represent another technique that can be applied for LiPON deposition. Different studies with varying plasma parameters revealed a maximum conductivity of 6 × 10^−7^ S cm^−1^.^[^ ^44^ ^]^

#### 3.1.2 Garnet

Li‐rich garnets, such as Li_5_La_3_Ta_2_O_12_ (LLTaO) and Li_7_La_3_Zr_2_O_12_ (LLZ), were first described by Thangadurai et al.^[^ ^45^ ^]^ and Murugan et al.,^[^ ^46^ ^]^ who demonstrated that these materials exhibit high bulk ionic conductivities in the range from 10^−6^ S cm^−1^ (LLTaO) to 10^−4^ S cm^−1^ (LLZ). LLZ forms either a cubic phase with high ionic conductivity (up to 1.8 × 10^−3^ S cm^−1[^ ^47^ ^]^) or a tetragonal phase with a conductivity of up to 2.3 × 10^−5^ S cm^−1^ depending on the composition and synthesis temperature.^[^ ^48^ ^]^ Various substitutions can be carried out while maintaining the garnet structure. The most common options are: the substitution of the Li‐site by Al^3+^ and Ga^3+^, the substitution of the La‐site by Ca^2+^, Sr^2+^, and Ba^2+^, and the substitution of the Zr/Ta‐site by Nb^5+^. Through these substitutions the amount of Li‐ion vacancies can be tuned precisely, and the cubic phase is stabilized. Thus, the ionic conductivity can be maximized. More details about substitutions are summarized in two review papers.^[^ ^49^ ^]^

Several attempts were made to deposit garnet structured materials by PVD processes. The proper selection of the deposition temperature in conjunction with the substrate temperature is a major challenge for the garnet deposition. Zr‐containing garnets are thermodynamically stable above 600–700 °C. At lower temperatures, amorphous phases, which exhibit low ionic conductivity, or La_2_Zr_2_O_7_ as the main phase were detected. At higher temperatures, a significant Li‐loss can occur due to the combination of vacuum, high temperature, and the unfavorable surface to volume ratio of thin films. The necessary temperature treatment (either during deposition or in a post‐annealing process) and the relatively high CTE of LLZ of about 15 × 10^−6^ K^−1[^ ^50^ ^]^ limit the choice of suitable substrate materials. Matching CTEs help avoiding thermomechanical tensile/compressive stresses and are therefore necessary to obtain thin films without cracks and spalls, which is essential for electrolyte layers.

The first publication that demonstrated the fabrication of a garnet structure with a reasonable ionic conductivity was based on the epitaxial deposition of LLZ by PLD on a Gd_3_Ga_5_O_12_ substrate, which itself exhibits a garnet structure.^[^ ^51^ ^]^ Further successful depositions via PLD were carried out on MgO substrates. Saccoccio et al. deposited Li_6.4_La_3_Zr_1.4_Ta_0.6_O_12_ at different temperatures followed by post‐annealing at 600 °C.^[^ ^52^ ^]^ A different approach consisted of depositing Li_6.25_Al_0.25_La_3_Zr_2_O_12_, alternating with Li_3_N layers to balance the Li‐loss during annealing, which was carried out at 660 °C.^[^ ^53^ ^]^

**Figure 5:**
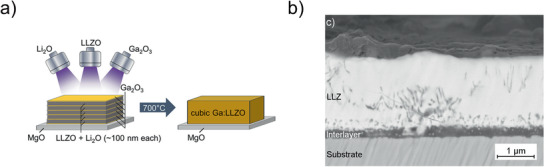
a) Multilayer deposition of Ga_2_O_3_ and co‐sputtered Li_2_O+LLZ layers which react to cubic LLZ after annealing at 700 °C. Reproduced with permission.^[^ ^54^ ^]^ Copyright 2018, American Chemical Society. b) Sputter deposition of garnet structured thin films on steel substrate at 700 °C leads to interlayer formation. Reproduced with permission.^[^ ^56^ ^]^ Copyright 2016, Elsevier.

A confocal sputtering approach was applied to deposit Ga‐ and Al‐substituted LLZ on MgO substrates.^[^ ^54^, ^55^ ^]^ Three targets, namely LLZ, Li_2_O and either Ga_2_O_3_ or Al were used, and a sequential deposition of single layers was performed (**Figure** **5**a). The thin films were deposited at room temperature and subsequently annealed at 700 °C to crystallize the garnet structure. Using sputter deposition from a Li_6.6_La_3_Zr_1.6_Ta_0.4_O_12_ target, LLZ thin films were also obtained on stainless steel substrates at a substrate temperature of 700 °C.^[^ ^56^ ^]^ In this case, a severe reaction between the thin film and the steel substrate was observed, which produced a Li—Al—O interfacial layer (Figure 5b). For both sputtering and PLD, a temperature between 600 °C and 700 °C is necessary during deposition or post‐annealing to obtain garnet structured LLZ. When a Zr‐free garnet structure is to be deposited, lower temperatures are sufficient for crystallization.^[^ ^57^, ^58^ ^]^ Reinacher et al. deposited Li_6_BaLa_2_Ta_2_O_12_ at 550 °C with a target that was enriched with 5 mol% Li_2_O to compensate for possible Li‐loss during deposition.^[^ ^57^ ^]^ The processes are also sensitive to the type of substrate. If inert material, such as MgO, is not used, reactions with the substrate are observed. Deposition on LiCoO_2_ (LCO) showed strong diffusion of Co into the garnet thin film.^[^ ^59^ ^]^ Therefore, a diffusion barrier had to be implemented.^[^ ^55^ ^]^

The ionic conductivity of PVD‐grown LLZ films with different orientation was 1.0 × 10^−5^ S cm^−1^ and 2.5 × 10^−6^ S cm^−1^ for the (111) and (100) orientation, respectively, with corresponding activation energies of 0.52 and 0.55 eV. These values are inferior to those of bulk LLZ, which is commonly observed for thin films, and could be explained by the loss of lithium during deposition.^[^ ^51^ ^]^ The highest conductivity of 1.2 × 10^−4^ S cm^−1^ was observed for a sputter deposition process at 700 °C.^[^ ^56^ ^]^ In general, the conductivity values of the thin films are lower than for comparable bulk material. It can be speculated that the relatively low crystallization temperatures prevent the crystallization of large grains and thus increase the overall grain boundary resistance.

Besides PVD, chemical vapor deposition (CVD) was applied successfully to deposit tetragonal LLZ as well as Zr‐free LLTaO with sufficiently high conductivities of 4.2 × 10^−6^ and 3.8 × 10^−5^ S cm^−1^, respectively.^[^ ^60^ ^]^ These values are close to those obtained with the bulk materials. LLZ thin films with a garnet structure synthesized by wet‐chemical methods, for example, sol–gel processes, have even lower conductivity values than thin films generated by PVD.^[^ ^61^, ^62^ ^]^ For example, the maximum value for a thin LLZ film deposited by a sol–gel process with subsequent annealing at 900 °C was 2.4 × 10^−6^ S cm^−1^.^[^ ^61^ ^]^

#### 3.1.3 NaSICON

The abbreviation NaSICON, which stands for Na‐Super‐Ionic‐CONductor, describes a class of compounds with a stable 3D framework consisting of two types of transition metal‐oxygen (MO_6_ and M'O_6_) octahedra that share all corners with either sulfate, phosphate, silicate or arsenate tetrahedra. The first NaSICON solid electrolytes with Na‐ion conductivity were described in 1976 by Goodenough^[^ ^63^ ^]^ and Hong.^[^ ^64^ ^]^ The same type of structure was reported later to also show a reasonable Li‐ion conductivity. Particularly, the materials with the composition Li_1+_ *_x_*Al*_x_*M_2‐_ *_x_*(PO_4_)_3_ (M = Ge,Ti) demonstrate Li‐ion conductivities as high as 7.4 × 10^−4^ S cm^−1^ for Li_1+_ *_x_*Al*_x_*Ti_2‐_ *_x_*(PO_4_)_3_ (LATP, *x* = 0.3)^[^ ^65^ ^]^ and 2.4 × 10^−4^ S cm^−1^ for Li_1+_ *_x_*Al*_x_*GeM_2‐_ *_x_*(PO_4_)_3_ (LAGP, *x* = 0.5).^[^ ^66^ ^]^ The lower sintering temperature of these materials compared to other ceramic electrolytes is an important advantage. Dense ceramics with high ionic conductivities can be obtained by sintering at 850 °C (LAGP)^[^ ^67^ ^]^ and 880 °C (LATP).^[^ ^68^ ^]^

A crystalline LAGP thin film was obtained by sputtering from a ceramic LAGP target at 500 and 600 °C. At lower temperatures a secondary phase consisting of AlPO_4_ was observed.^[^ ^69^ ^]^ Tan et al. deposited Li—Al—Ti—P—O—N thin films in a temperature range between 25 and 500 °C.^[^ ^70^ ^]^ The as‐deposited films were amorphous with an increasing fraction of nanocrystalline domains in response to increasing deposition temperature. The ionic conductivities improved with increasing substrate temperature, showing a maximum of about 1.2 × 10^−5^ S cm^−1^ at 30 °C for a sample deposited at 500 °C with simultaneous reduction of the activation energy from 0.63 eV (deposition at 25 °C) to 0.44 eV. The Li‐ion transference number was calculated to be 0.9999 based on a polarization measurement. Hebb–Wagner measurements showed a low electronic conductivity in the range of 7.5 × 10^−12^ to 4.9 × 10^−11^ S cm^−1^.^[^ ^70^ ^]^ The thin film was applied in a battery consisting of a NMC111 cathode and a metallic lithium anode, which showed a discharge capacity of about 127 mAh g^−1^ after 100 cycles.^[^ ^71^ ^]^

Recently, Hofmann et al. studied the crystallization behavior of LATP deposited by a PLD process. The deposition was first carried out on different substrates, namely, MgO, YSZ, Al_2_O_3_, and n‐doped Si, among which n‐doped Si showed the lowest amount of secondary phases. In a second step the deposition at elevated temperatures as well as the post‐crystallization of films deposited without heating were investigated. High temperature X‐ray diffraction studies revealed the formation of the NaSICON phase between 650 and 825 °C. Further, secondary phases were observed at 850 °C due to element interdiffusion between the substrate and the thin film. Crystallization studies of amorphous films at 750 °C showed that only short dwell times were necessary for the formation of the NaSICON structure.^[^ ^72^ ^]^

#### 3.1.4 Perovskites

The perovskite Li_3_ *_x_*La_2/3‐_ *_x_*_‐1/3‐2_ *_x_*TiO_3_ (LLT, *x* = 0.11), which was discovered by Inaguma et al., shows a high bulk conductivity of 1 × 10^−3^ S cm^−1^, but the total conductivity of this material is typically lower due to the low grain boundary conductivity of 7.5 × 10^−5^ S cm^−1^.^[^ ^73^ ^]^ The bulk conductivity of the perovskite structure can be tuned via substitution, for example, by Sr on an A(Li/La)‐site or by Al, Ta, Zr, Nb on a B‐Site(Ti).^[^ ^74^ ^]^

PVD of LLT was mainly carried out by PLD processes,^[^ ^75^, ^76^, ^77^, ^78^, ^79^ ^]^ whereas only a few publications focused on sputtering^[^ ^80^, ^81^ ^]^ or evaporation processes.^[^ ^82^ ^]^

The deposition temperature is a crucial parameter for the conductivity of LLT thin films deposited by PLD. Below 700 °C, either during deposition or annealing, the thin films remain in an amorphous state, enabling higher ionic conductivities due to the absence of grain boundaries. Lee and Ahn reported 400 °C as an ideal deposition temperature.^[^ ^76^, ^79^ ^]^ Post‐annealing after deposition at 500 °C did not show a significant improvement of the ionic conductivity.^[^ ^75^ ^]^ Controlling the oxygen partial pressure and the deposition temperature led to ionic conductivities of up to 3.0 × 10^−4^ S cm^−1^ at room temperature.^[^ ^79^ ^]^ Furusawa et al. investigated the ionic conductivity for the composition Li*_x_*La_(2‐_ *_x_*_)/3_TiO_3_ while varying the Li‐content (0.1 ≤ *x* ≤ 0.5) and the crystallinity. Amorphous thin films demonstrated higher ionic conductivities than the comparable polycrystalline samples, reaching up to 1.252 × 10^−3^ S cm^−1^ for *x* = 0.5.^[^ ^78^ ^]^ In accordance with the temperature range for amorphous thin films, epitaxial layers of LLT were deposited on SrTiO_3_ (STO) single crystals at substrate temperatures of 800 °C and above.^[^ ^77^ ^]^ The conductivity of these films was 5.63 × 10^−5^ S cm^−1^ at room temperature for STO(111).^[^ ^77^ ^]^ Sputter deposited films show a perovskite main phase with an increasing amount of secondary phases with increasing deposition temperature and a total conductivity of 5.25 × 10^−5^ S cm^−1^.^[^ ^80^ ^]^ Films deposited by electron beam evaporation exhibited an ionic conductivity of 1.8 × 10^−7^ S cm^−1^ for the as‐deposited phase.^[^ ^82^ ^]^ Post‐annealing at 100 °C increased the resistance of the thin film.^[^ ^82^ ^]^

### 3.2 Deposition and Properties of Thin Film Electrodes

In this chapter the discussion of the processing‐property relationships of electrode active materials is focused on materials that were already published in the context of all‐solid‐state batteries. Reviews about thin films for battery components deposited by different techniques (not exclusively by PVD)^[^ ^2^ ^]^ as well as by PLD^[^ ^83^ ^]^ are recommended for further reading.

#### 3.2.1 Intercalation Materials

The most important group of electrodes are the intercalation materials. In state‐of‐the‐art batteries both electrodes typically include intercalation‐type active materials, such as graphite and Li_4_Ti_5_O_12_ (LTO) for the negative electrodes and LCO and LiFePO_4_ (LFP) for the positive electrodes. For most intercalation electrodes, the crystallization behavior of the layers is different for PLD and sputter processes, because the electrochemical properties of the materials are dependent on the oxidation state of the transition metal ions. With PLD higher oxygen partial pressures can be realized, and therefore the crystallization of the material is usually achieved by in situ heating during deposition, whereas sputter‐processes are typically carried out without active heating, and crystallization occurs during a post‐annealing step.

##### Electrode Materials with a Layered Structure

Layered materials with a general composition of Li(Ni*_x_*Mn*_y_*Co*_z_*)O_2_ (NMC, with *x*+*y*+*z* = 1) are commonly used cathode active materials (CAMs) in state‐of‐the‐art lithium‐ion batteries. The most investigated material of this family is LCO, which crystallizes analogously to a layered *α*‐NaFeO_2_‐structure, and has a theoretical capacity of about 148 mAh g^−1^ (about 70 μAh cm^−2^ µm^−1^) and a potential of about 3.8 V vs Li/Li^+^.^[^ ^84^ ^]^ Due to the importance of LCO as a CAM in commercial batteries, it attracted an immense research interest including PVD processes, which have been investigated for almost 30 years. A detailed overview of sputter deposition and PLD of LCO thin films is given in the publication put forward by Julien et al.^[^ ^83^, ^85^ ^]^ Therefore, only the most important and most recent developments will be reviewed in detail in this work.

By applying PLD, crystalline LCO films with satisfactory electrochemical properties, i.e., high discharge capacities and stable cycling behavior, can be obtained at temperatures of 600 °C.^[^ ^86^, ^87^ ^]^ An oxygen pressure of about 6.7 Pa during the deposition process provided films with a discharge capacity of about 57 μAh cm^−2^ µm^−1^ after 20 cycles, while higher pressures of about 40 Pa were found to be detrimental for the electrochemical performance due to an unfavorable crystal orientation.^[^ ^87^ ^]^ LCO thin films deposited by sputter deposition showed the best electrochemical performance, as indicated by sufficient long‐term stability during cycling when post‐annealed at 700 °C.^[^ ^88^ ^]^

Crystalline LCO films can be obtained by sputtering processes at even lower temperatures if a bias voltage is applied to enhance the crystallinity, as was successfully demonstrated for LCO films deposited on aluminum substrates.^[^ ^89^ ^]^ Cells with liquid as well as solid‐state electrolyte showed discharge capacities of about 50 μAh cm^−2^ µm^−1^ after a post‐annealing step at 500 °C.^[^ ^89^ ^]^ This value is lower than for high temperature‐crystallized LCO thin films. The high values obtained with LCO thin films crystallized at about 700 °C could also not be achieved with other low‐temperature techniques.^[^ ^90^ ^]^

Since LCO has a layered structure, its ionic conduction is highly anisotropic and it depends on the orientation of the deposited layer. For LCO deposited on Pt‐ and Au‐coated alumina substrates Bates et al. observed that the orientation is changing from the unfavorable (003) to the more favorable (101) orientation with increasing layer thickness.^[^ ^91^ ^]^ This assumption is rationalized by a minimization of the surface energy for very thin films and a minimization of volume strain for thicker films.^[^ ^91^ ^]^ However, this effect depends strongly on the substrate material.^[^ ^92^ ^]^ The film orientation can also be controlled by the deposition of epitaxial LCO, which can be obtained by PLD processes with substrates made of STO,^[^ ^93^ ^]^ (110)Pt,^[^ ^94^, ^95^ ^]^ and (110)Au,^[^ ^95^ ^]^ respectively.

**Figure 6:**
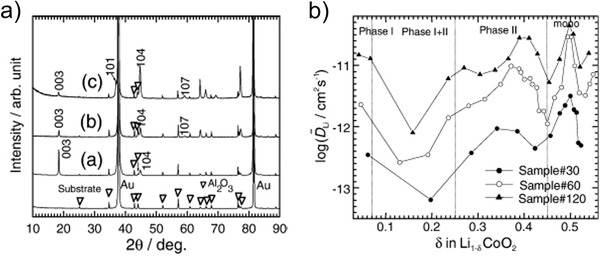
a) Change of preferred orientation of LCO thin films deposited for 30 min (denoted as (a), thickness ≈0.31 µm), 60 min (denoted as (b), thickness ≈0.77 µm), and 120 min (denoted as (c), thickness ≈1.35 µm) and b) correlated diffusion coefficients as a function of the amount of Li intercalated into the LCO structure as determined by GITT. Reproduced with permission.^[^ ^96^ ^]^ Copyright 2008, Elsevier.

Orientation‐dependent Li^+^ diffusion coefficients were determined for LCO thin films deposited on Au‐coated alumina with thicknesses between 0.31 and 1.35 µm.^[^ ^96^ ^]^ In agreement with the work of Bates et al.,^[^ ^91^ ^]^ the orientations of the thin films were dependent on the film thickness, showing a (003) orientation and (104) orientation for the thinner and thicker films, respectively (**Figure** **6**a). The diffusion coefficients were determined via different techniques, namely galvanostatic intermittent titration technique (GITT), potentiostatic intermittent titration technique, electrochemical impedance spectroscopy, and cyclic voltammetry (CV). GITT measurements revealed that the Li‐ion diffusion was most favorable for the (104) oriented thin film (Figure 6b).^[^ ^38^, ^39^ ^]^

**Figure 7:**
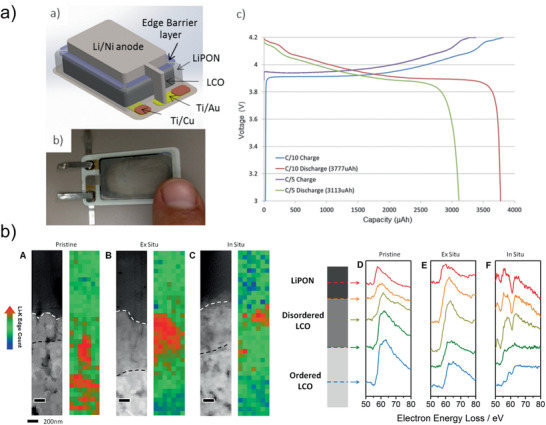
a) Illustration, photograph, and electrochemical cycling data for a large‐scale thin‐film battery with a thick LiCoO_2_ electrode. Reproduced with permission.^[^ ^97^ ^]^ Copyright 2017, Elsevier. b) In situ TEM showing degradation of LiCoO_2_ thin film in an all‐solid‐state battery during cycling at elevated temperatures. Reproduced with permission.^[^ ^101^ ^]^ Copyright 2016, American Chemical Society.

Thicker LCO films are required to increase the total capacity of thin‐film batteries. All‐solid‐state cells with a 10 µm thick LCO cathode obtained by sputter deposition showed high discharge capacities of about 45 μAh cm^−2^ µm^−1^ at a discharge rate of 1 C. Increasing the cell footprint to an area of 5.89 cm^2^ yielded a total discharge capacity of 3777 μAh (**Figure** **7**a).^[^ ^97^ ^]^ However, in a similar study with thin‐film batteries deposited by PLD cracks were observed in films with a thickness of about 12.5 µm after cycling.^[^ ^98^ ^]^

LCO‐based thin‐film batteries showed strong degradation during cycling at elevated temperatures.^[^ ^99^ ^]^ For example, while the discharge capacity remained very stable over 140 cycles at 25 and 50 °C, a ≈20% and ≈40% loss was observed at 100 and 150 °C, respectively. This can be explained by the formation of a disordered LCO phase at the LCO/LiPON interphase.^[^ ^100^, ^101^ ^]^ In situ STEM‐EELS indicated that the phase consisted of Li_2_O and CoO, which formed during LCO decomposition (Figure 7b).^[^ ^101^ ^]^ This layer grew to a thickness of 4 µm after 250 cycles at 80 °C,^[^ ^100^ ^]^ which explains the rapid degradation of thin‐film batteries at such elevated temperatures.

Layered NMC materials in which Co is substituted by Ni and/or Mn in the *α*‐NaFeO_2_ structure gradually replace LCO as CAMs due to their higher capacities (e.g., 160 mAh g^−1^ for LiNi_1/3_Mn_1/3_Co_1/3_O_2_^[^ ^84^ ^]^) and gain increasing importance also in PVD research.

PLD of NMC thin films was reported by several groups. The oxygen partial pressure and the applied deposition temperature were varied in a very broad range from 3.3 to 266 Pa.^[^ ^102^, ^103^ ^]^ The applied deposition temperature ranged from 450 °C to 750 °C to achieve crystalline thin films,^[^ ^102^, ^103^ ^]^ whereby temperatures of 700 °C and higher were required to achieve high discharge capacities and low degradation during cycling.

For sputter deposition processes for the fabrication of NMC thin films, the temperature for post‐annealing typically ranges from 400 to 800 °C, showing improved electrochemical properties at higher temperatures,^[^ ^71^, ^104^, ^105^, ^106^ ^]^ whereas the poorly crystallized phase showed fast deterioration of the discharge capacity. An approximate capacity loss of 80% over 200 cycles at 50 µA µm^−1^ cm^−2^ was observed in a half‐cell configuration.^[^ ^107^ ^]^ However, thin films were Li‐deficient after post‐annealing in those cases where a Li‐excess in the sputter target was not provided.^[^ ^105^, ^106^ ^]^ Nevertheless, even for Li‐deficient thin films high initial discharge capacities of up to 202 mAh g^−1^ were observed for NMC111 during cycling with liquid electrolyte between 2.8 V and 4.5 V.^[^ ^106^ ^]^

##### Electrodes with a Spinel Structure

The manganese spinel LiMn_2_O_4_ (LMO) is an attractive cathode material due to the abundance and non‐toxicity of the composing elements. The theoretical capacity of LMO is 148 mAh g^−1^. However, only 100–120 mAh g^−1^ can be practically realized because of the structural instability due to the Jahn–Teller distortion.^[^ ^84^ ^]^

The Li—Mn—O phase diagram is quite complex. Different compositions are possible depending on the deposition conditions.^[^ ^108^, ^109^ ^]^ The range of deposition parameters is quite large in comparison to other intercalation electrodes. PLD is carried out at temperatures between 300 and 650 °C and oxygen pressures between 2.66 and 30 Pa.^[^ ^110^ ^]^

LMO electrodes were sputter‐deposited without active heating, followed by post‐annealing between 600 and 750 °C.^[^ ^111^, ^112^, ^113^ ^]^ Dudney et al. showed that amorphous and nanocrystalline thin films are Mn‐deficient and that the degree of deficiency is dependent on the oxygen partial pressure.^[^ ^108^ ^]^ It is remarkable that the amorphous layers partly yield higher discharge capacities in all‐solid‐state batteries compared to the crystallized phase.^[^ ^108^ ^]^ At cell potentials above 3 V a capacity of 100–150 mAh g^−1^ was observed for the amorphous material, whereas stoichiometrically comparable crystalline compositions yielded 20–50 mAh g^−1^ at best.

Substitution of manganese by nickel in the LMO structure introduces a high‐voltage Ni^2+^/Ni^4+^ redox couple with the potential of 4.7 V,^[^ ^114^ ^]^ resulting in a high‐voltage LiNi_0.5_Mn_1.5_O_4_ (LNMO) cathode material. Therefore, the deposition and the properties of LNMO thin films have been the focus of several publications.

**Figure 8:**
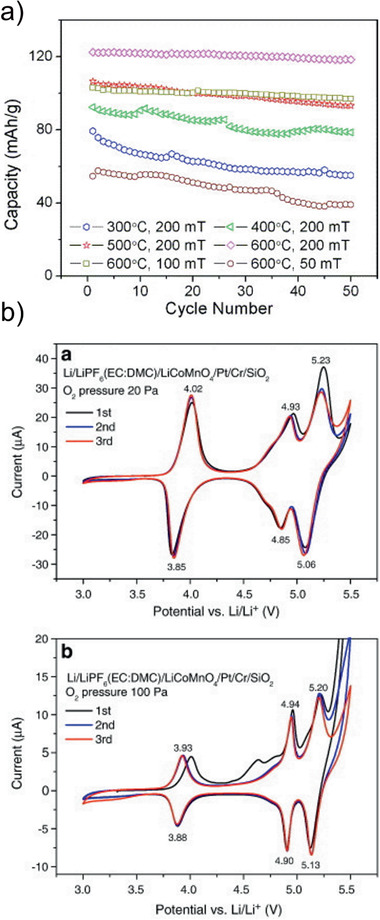
a) Discharge capacity of LNMO deposited by PLD thin films as a function of the deposition parameters. Cycled with 1 m LiPF_6_ in EC/DEC (1:1) and Li‐counter electrode between 3 and 5 V. Reproduced with permission.^[^ ^115^ ^]^ Copyright 2007, Elsevier. b) Cyclic voltammograms of LCMO thin films cycled with 1 m LiPF_6_ in EC/DEC (1:1) with a Li counter electrode at a sweep rate of 0.5 mV s^−1^. Reproduced with permission.^[^ ^116^ ^]^ Copyright 2014, Elsevier.

The only systematic study of LNMO deposition by PLD was performed by Xia et al. who demonstrated that an oxygen pressure of about 26.6 Pa and deposition temperature of 600 °C are the optimal process parameters, resulting in films with high initial capacities of 122.5 mAh g^−1^ for the first cycle and a capacity retention of 96% after 50 cycles in liquid electrolyte cells (**Figure** **8**a).^[^ ^115^ ^]^

LNMO films can be also obtained via sputter deposition. However, the layers deposited at low temperature exhibit poor crystallinity, and have to be heated during deposition or post annealed to increase the crystallinity. The choice of the processing parameters is thereby highly dependent on the substrate. If inert substrates, such as Pt/Al_2_O_3_ and Pt/Si, were used, the LNMO films can be deposited without heating, and can be post annealed at temperatures of about 700–750 °C, resulting in electrode layers with satisfactory electrochemical properties.^[^ ^117^, ^118^ ^]^ If stainless steel was used as a substrate, the annealing temperatures would have to be lowered to about 500–600 °C to avoid the substrate oxidation.^[^ ^119^, ^120^ ^]^ These temperature are, however, not sufficient for the LNMO crystallization, as the samples annealed at 550 °C still show relatively low capacities (<100 mAh g^−1^) and strong degradation, as evident from an observed discharge capacity loss of 53.1% over 150 cycles.^[^ ^120^ ^]^

Li‐content variation in the sputter target revealed that a stoichiometric target leads to the formation of Li_2_MnO_3_ and LiMnO_2_ in the resulting thin film, whereas the thin films deposited from a target with low Li‐content (Li_0.5_Ni_0.5_Mn_1.5_O_4_) show low discharge capacities.^[^ ^117^ ^]^ The highest discharge capacity of about 58 μAh cm^−2^ µm^−1^ during cycling with liquid electrolyte was obtained for a film deposited with a Li_0.75_Ni_0.5_Mn_1.__5_O_4_ sputter target with reduced Li‐content.^[^ ^117^ ^]^

Cobalt‐substitution in LMO leads to a 5 V cathode material due to the Co^3+^/Co^4+^ redox couple.^[^ ^121^ ^]^ LiCoMnO_4_ (LCMO) thin films were deposited by PLD between 500 °C and 700 °C.^[^ ^116^, ^122^, ^123^ ^]^ A lower oxygen deficiency in the structure was reported as a consequence of increased oxygen pressure. Optimal deposition pressures of 100–200 Pa were suggested (Figure 8b).^[^ ^116^ ^]^ LCMO films deposited at lower oxygen pressures of 6.6 Pa showed stronger degradation during electrochemical cycling, specifically, a discharge capacity loss of approximately 30% over 20 cycles, compared to films deposited at 100 Pa.^[^ ^123^ ^]^ While films obtained at higher oxygen partial pressures showed a more distinct high‐voltage behavior in liquid electrolytes, higher discharge capacities in all‐solid‐state batteries with a Li anode and a LiPON electrolyte were observed for LCMO films deposited at 20 Pa.^[^ ^116^ ^]^ LCMO thin films reported in another publication and cycled with lower cut‐off voltages exhibited remarkably high discharge capacities of 340 mAh g^−1^ (cycling with liquid electrolyte between 1.5 V and 4.5 V)^[^ ^123^ ^]^ and 362 mAh g^−1^ (cycling in all‐solid‐state battery with a Li anode and a LiPON electrolyte between 1.4 V and 5 V).^[^ ^122^ ^]^

##### Other Active Electrode Material Structures

Olivine‐structured lithium transition‐metal phosphates LiMPO_4_ (M = Fe, Mn, Co, Ni) are a popular class of cathode materials with a capacity of about 170 mAh g^−1^ and high stability during electrochemical cycling. Particularly, LiFePO_4_ (LFP) with an operating potential of 3.45 V is a commercial low‐cost CAM with very high chemical and electrochemical stability. The operation potential and therefore the energy density of olivine CAMs increase as follows: Fe < Mn < Co < Ni. The major drawback of all olivine materials is their low electronic and ionic conductivity. An electronic conductivity of ≈10^−9^ S cm^−1[^ ^124^ ^]^ and Li^+^ diffusion coefficients of about 10^−13^–10^−14^ cm^2^ s^−1^ were reported for LFP applying different measurement techniques.^[^ ^125^ ^]^

**Figure 9:**
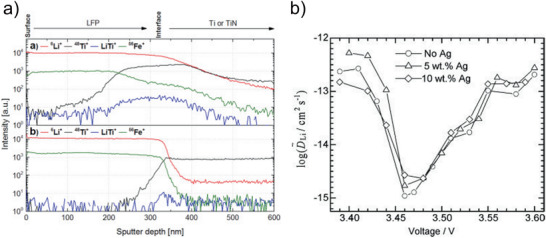
a) SIMS depth profile of LFP deposited on metallic Ti showing strong interdiffusion of Ti into the LFP thin film (top) and of suppressed Ti‐diffusion by implementation of a TiN diffusion barrier layer. Reproduced with permission.^[^ ^131^ ^]^ Copyright 2015, Elsevier. b) Diffusion coefficient for Li^+^ determined by EIS in LFP thin films with varying Ag content as function of voltage. Reproduced with permission.^[^ ^128^ ^]^ Copyright 2009, Elsevier.

To avoid the oxidation of Fe^2+^, LFP thin films have to be crystallized in an atmosphere with low oxygen pressure, which is typically achieved by a post‐annealing step for both sputtering and PLD. The formation of crystalline olivine phases with satisfactory electrochemical properties was reported for annealing temperatures ranging from 500 to 700 °C.^[^ ^126^, ^127^, ^128^ ^]^ Metallic iron precipitates on the surface of LFP thin films after the crystallization step were observed for MgO,^[^ ^129^ ^]^ Si^[^ ^130^ ^]^ and Ti^[^ ^131^ ^]^ substrates. In the latter case, SIMS measurements revealed an interdiffusion of Ti into the LFP layer, which probably led to the formation of the precipitates. The application of a TiN layer as a diffusion barrier enabled the deposition of pure LFP thin films (**Figure** **9**a).^[^ ^131^ ^]^

The capacity utilization of Li in pure LFP thin films is typically very poor. To date, only Yada et al. reported the formation of a thin LFP film by PLD with a capacity that is close to the theoretical value without any conductive additives present.^[^ ^127^ ^]^ However, the film thickness was only 50 nm, and the total capacity achieved during cycling in liquid electrolyte between 3.0 and 4.0 V was only about 0.4 μAh. The introduction of conductive species like carbon or silver into the electrode layers can increase the electronic conductivity of LFP and improve its cycling behavior. For sputter deposition processes this approach was realized by using carbon‐ or silver‐containing deposition targets. Thin films deposited by sputter deposition from C‐rich targets showed only a small temperature window for single phase LFP due to secondary phase formation. Post‐annealing at 500 °C yielded a minor amount of secondary phases leading to discharge capacities of up to 170 mAh g^−1^ depending on other deposition parameters, for example, substrate bias.^[^ ^132^ ^]^ Sputter deposition at 600 °C led to the formation of additional phases besides LFP due to a carbothermal reaction.^[^ ^133^ ^]^ As a result of this reaction a 3D structure was formed, and the capacity was greatly increased to 160 mAh g^−1^.^[^ ^133^ ^]^

The introduction of silver enables a higher tolerance with respect to the annealing temperature. LFP thin films were obtained without secondary phases by performing PLD at 600 °C^[^ ^134^ ^]^ and by sputter deposition and subsequent annealing at 700 °C for 1 h in Ar/H_2_.^[^ ^128^ ^]^ The samples that were deposited by PLD showed discharge capacities that were close to the theoretical limit when the cut‐off voltages were extended to the range from 2.5 to 4.5 V.^[^ ^134^ ^]^ The thin films deposited by sputtering showed an increase in the discharge capacity from 30 to 60 mAh g^−1^ due to the addition of 10 wt% silver.^[^ ^128^ ^]^ The diffusion coefficients determined for these samples were nearly independent of the silver content (Figure 9b).^[^ ^128^ ^]^

The high‐voltage material LiCoPO_4_ showed low discharge capacities of less than 12 μAh cm^−2^ µm^−1^ in the form of phase pure thin films.^[^ ^135^ ^]^ LiMnPO_4_ was deposited by PLD at varying substrate temperatures and deposition pressures, yielding the highest discharge currents for the films deposited at a substrate temperature of 600 °C and 100 Pa Ar. The highest reported capacities, which were determined in a thin‐film cell with a LiPON electrolyte and a Li anode, were 28 ± 3 mAh g^−1^, which is 16% of the theoretical capacity.^[^ ^136^ ^]^ The low capacity was explained by the low Li^+^ diffusion coefficient of 3 × 10^−17^ cm^2^ s^−1^ as determined by CV.^[^ ^136^ ^]^

The zero‐strain material Li_4_Ti_5_O_12_ (LTO) has a spinel structure and a theoretical capacity of about 175 and 293 mAh g^−1^ corresponding to the intercalation of 3 or 5 Li‐ions, respectively.^[^ ^137^ ^]^ For PLD processes, 700 °C was determined as the ideal deposition temperature to obtain well‐crystallized thin films.^[^ ^138^ ^]^ Phase pure LTO was formed at oxygen pressures of 1.3 × 10^−2^ Pa^[^ ^139^ ^]^ and 3 Pa,^[^ ^140^ ^]^ whereas the formation of LiTi_2_O_4_ took place at 1.3 × 10^−4^ Pa,^[^ ^139^ ^]^ and secondary phases were formed at 6 Pa and above.^[^ ^140^ ^]^ A full all‐solid‐state battery with a LTO thin‐film cathode deposited by PLD on a LLZ pellet at 500 °C and a Li‐foil anode was cycled at low C‐rates (highest C‐rate = 0.15C), and showed discharge capacities close to the theoretical value of LTO at 0.015C.^[^ ^141^ ^]^

Sputter deposition was mainly carried out without substrate heating and subsequent annealing of the samples. Temperatures of about 1000–1100 °C yielded the formation of TiO_2_ (rutile structure) secondary phases,^[^ ^142^ ^]^ whereas at lower temperatures pure LTO was identified.^[^ ^142^, ^143^, ^144^ ^]^ The addition of Li_2_O during sputtering increased the amount of Li in the film after annealing.^[^ ^143^ ^]^

Deposition by ion beam sputtering yielded the highest crystallinity when the film was deposited at 600 °C and an oxygen partial pressure of 3 × 10^−4^ mbar was employed.^[^ ^144^ ^]^ Li‐diffusion coefficients were determined on these films by CV, galvanostatic potentiometry and GITT.^[^ ^144^ ^]^ All values were in the range of 10^−12^ cm^2^ s^−1^, which is in agreement with the values obtained with NMR measurements on powder samples.^[^ ^145^ ^]^

MoO_3_ and V_2_O_5_ are electrode materials with high capacities of up to 280 and 147 mAh g^−1^, respectively. Due to their intermediate redox potential, they can be applied either as positive or negative electrode materials in different types of batteries. Both materials are very attractive as electrodes for thin film batteries, because a crystallization of the phase is not necessary, and therefore, the deposition can be carried out on polymer substrates as well.

MoO_3_ cathodes for thin film batteries were sputtered either from MoO_3_ targets^[^ ^146^, ^147^ ^]^ or by reactive sputtering with oxygen from a Mo target.^[^ ^148^ ^]^ The resulting all‐solid‐state batteries showed good cycling behavior even with thicker electrodes. A 4.66 µm thick film allowed for the utilization of 81.7% of its volumetric discharge capacity relative to an otherwise identical 1 µm thick film.^[^ ^147^ ^]^ For comparison, similar cells with LCO cathodes rapidly deteriorated, losing 50% of the initial capacity within 100 cycles at an elevated temperature of 150 °C, whereas a stable capacity for more than 10000 cycles were achieved with a MoO_3_ cathode.^[^ ^146^ ^]^

**Figure 10:**
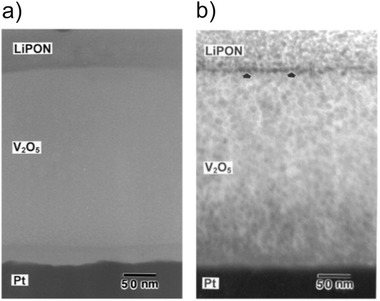
Cross section bright field TEM of the cathode part of a V_2_O_5_/LiPON/Li battery a) as deposited b) after 450 charge/discharge cycles. The initially amorphous phases V_2_O_5_ and LiPON show signs of crystallization after electrochemical cycling. Reproduced with permission.^[^ ^149^ ^]^ Copyright 2003, American Vacuum Society.

V_2_O_5_ cathodes for thin‐film batteries were deposited either by reactive sputtering with a vanadium target in the presence of oxygen^[^ ^149^, ^150^ ^]^ or by thermal evaporation of V_2_O_5_.^[^ ^151^, ^152^ ^]^ Aside from the commonly used Pt‐coated silicon wafer substrate,^[^ ^149^ ^]^ the deposition was also carried out on stainless steel^[^ ^150^, ^152^ ^]^ as well as on flexible aluminum substrates.^[^ ^151^ ^]^ Structural analysis of a V_2_O_5_/LiPON/Li battery after 450 cycles at room temperature revealed the formation of nanocrystals on both the V_2_O_5_ cathode and the LiPON electrolyte (**Figure** **10**).^[^ ^149^ ^]^

#### 3.2.2 Alloy Electrodes

Materials that are able to store lithium via an alloying mechanism are the subject of intensive research activities, as they can function as anodes with a high energy density for future battery concepts including solid‐state batteries. Currently, the most frequently used anode in all‐solid‐state batteries is made of Li metal, which is applied in thin‐film batteries by a vapor deposition process.^[^ ^153^ ^]^ However, details about the vapor deposition process are rarely reported, and the influence of the Li‐anode application in thin‐film cells has not been discussed in the literature yet. For bulk‐type batteries, the Li metal anode is usually applied as a foil. Details about the interface between Li metal and solid electrolytes are given in Section 4.2.

Silicon, tin and germanium are the most popular alloying anode materials due to their availability, relative stability, high theoretical capacity and low potential (4200 mAh g^−1^/0.4 V, 994 mAh g^−1^/0.6 V and 1625 mAh g^−1^/0.5 V for Si, Sn, and Ge, respectively).^[^ ^154^ ^]^ These materials have also been investigated in combination with solid electrolytes as buffer layers between the electrolyte and the Li metal anode.^[^ ^155^, ^156^, ^157^, ^158^ ^]^

As in part determined by their melting temperature, thin films deposited by sputtering or evaporation without active substrate heating are amorphous in the case of Si und Ge and crystalline in the case of Sn. All materials show a large volume variation during electrochemical cycling (Si: 420%, Ge: 370%, Sn: 260%).^[^ ^154^ ^]^

Silicon electrodes show large irreversible capacity losses of roughly 500–1000 mAh g^−1^ during the first 5–10 cycles,^[^ ^159^, ^160^ ^]^ which can be attributed to their substantial expansion during lithiation and the associated loss of contact in the electrode. Similar observations were reported for Ge electrodes, albeit with smaller relative losses.^[^ ^161^ ^]^ The expansion effect becomes more severe with increasing film thickness for both materials.^[^ ^160^, ^161^ ^]^ Roughening thin films either by using a rough substrate^[^ ^162^ ^]^ or by nanostructuring^[^ ^163^ ^]^ could counteract the degradation during cycling test to a certain degree. For further reading a recently published review about silicon thin films as battery anodes is recommended.^[^ ^164^ ^]^

Silicon films deposited by sputter deposition were investigated as anodes in thin‐film all‐solid‐state batteries.^[^ ^157^, ^158^ ^]^ Thin‐film cells with a lithium anode, a B‐doped LiPON electrolyte and a thin silicon cathode exhibited a reversible capacity of about 571 μAh cm^−2^ µm^−1^ for 1500 cycles when cycled in an all‐solid‐state battery with a lithium anode and a B‐doped LiPON electrolyte between 0.05 and 1 V.^[^ ^158^ ^]^ Microbatteries with an amorphous lithium titanium oxysulfide cathode, a LiPON electrolyte and a silicon anode showed a mean capacity fade of 0.015% per cycle over 200 cycles.^[^ ^157^ ^]^

Similarly to Si, pure Sn thin films also suffered high capacity losses during the first cycles, as more than 20% of the initial capacity faded during the first 10 cycles.^[^ ^165^ ^]^ To optimize the performance of the alloying electrodes and to identify materials with an increased cycling stability, Dahn et al. investigated numerous binary allows of Si and Sn by combinatorial sputtering using the sputtering system described in Figure 3c. These materials were tested in liquid electrolyte cells and are shown here to demonstrate the potential for application of PVD to generate material libraries. For the Si_1‐_ *_x_*Sn*_x_* system the researchers identified compositions with high silicon content demonstrating a discharge capacity of up to 3500 mAh g^−1^ and reduced irreversible capacity losses (<15%). At low Sn concentrations the alloy remains amorphous during charge and discharge and there is no tendency toward particle growth for higher Sn‐contents.^[^ ^166^ ^]^

Binary Si‐Ag^[^ ^167^ ^]^ and Si‐Zn^[^ ^168^ ^]^ systems were investigated as alloy anodes by the same approach using thin‐film material libraries. During operation, a different behavior was identified for Ag compared to Zn. Ag was found to segregate into domains with an increasing size during cycling, thus severely compromising the initially alloyed structure. In contrast, Zn formed a dimensionally stable nanocrystalline dispersion in silicon, which acted as a confining matrix, and thus the degradation of silicon due to its volume changes was minimized. However, a continuous amorphous phase that does not undergo crystallization processes, as it is found in Sn‐Si, was identified as the most beneficial alloy anode.^[^ ^169^ ^]^

A similar principle of adding an electrochemically inactive element to generate a mechanically stable and chemically inert backbone was also investigated by means of thin‐film libraries for the following systems: Si—Fe,^[^ ^170^, ^171^ ^]^ Si—Co,^[^ ^172^ ^]^ Si—B,^[^ ^173^ ^]^ Si—M (M = Cr+Ni, Fe, Mn),^[^ ^174^ ^]^ and Si—Ni.^[^ ^175^ ^]^ For Si—Co,^[^ ^172^ ^]^ Si—Fe, Si—Mn, and Si—Cr+Ni thin layer electrodes capacity fading was observed,^[^ ^174^ ^]^ which was attributed to the formation of inactive silicides due to complete electrochemical inactivity at a concentration of 50 at% Si. The formation of inactive FeSi_2_ was confirmed also in subsequent work.^[^ ^170^ ^]^ The capacity drop observed for the Si—Ni system was attributed to the voltage suppression due to internal stress–voltage coupling.^[^ ^175^ ^]^ In the Si—B system neither the formation of detrimental phases nor a capacity drop was observed, and in contrast to the transition metal systems, only a minor voltage shift due to voltage‐stress coupling was detected.

Tin‐containing anodes with a general composition of Sn—M—C (M = Co, Ti, V) were also investigated by means of material libraries generated by sputter deposition. The selected materials showed an amorphous region in combination with tin, whereas other transition metals, such as Cr, Mn, Fe, Ni, and Cu formed crystalline phases.^[^ ^176^ ^]^ Detailed investigations of the ternary systems with carbon showed that only Sn—Co—C is a suitable candidate for advanced battery anodes, because transition metal carbides cannot be formed. Such a formation would lead to a detrimental phase separation of tin.^[^ ^177^ ^]^ Initial investigations showed that in (Sn_0.55_Co_0.45_)_1‐_ *_y_*C*_y_* for *y* = 0.4 a stable amorphous phase with a capacity of about 700 mAh g^−1^ was formed, which remained amorphous during electrochemical cycling.^[^ ^178^ ^]^ Further studies revealed stable cycling behavior also for similar phases, which were synthesized by mechanical methods as opposed to sputtering, but yielded lower specific capacities of 270 or 450 mAh g^−1^. This behavior was attributed to different particle sizes obtained by different synthesis methods.^[^ ^179^ ^]^

#### 3.2.3 Conversion‐Type Electrode Materials

Conversion materials have the general formula M*_x_*A*_y_* where M is a metal, mainly from the group of transition metals (Ti, V, Cr, Mn, Fe, Co, Ni, Sn, Zn, …) and A is an anion, mainly from the group O, N, F, S, P. As indicated by the name, conversion materials undergo the full conversion of the metal from oxidized to its metallic state (M*_x_*A*_y_* + (*y* × n) Li^+^ → *x* M + *y* Li*_n_*A). Such transformations often accommodate more than one Li atom per metal atom, resulting in very high capacities and energy densities. A general overview about conversion anodes can be found in the publication by Cabana et al.,^[^ ^180^ ^]^ and a general review about nitrides and carbides for energy conversion was published by Zhong et al.^[^ ^181^ ^]^

Various PVD techniques have been employed to deposit thin layers of conversion materials either as battery components or as model systems to investigate the mechanism of the material transformation during operation. PVD processing of conversion electrodes is mainly applicable for oxides and nitrides due to the fact that these types of materials can be easily deposited by reactive processes from metallic targets.

The main drawbacks of conversion materials are the significant chemical reorganization during reactions and generally poor ionic and electronic conductivity. The consequence is a substantial potential hysteresis and poor capacity retention, both hampering the application of these materials as anodes. Understanding the mechanisms of underlying material transformations and finding ways to improve the electrode performance has been one important task in PVD‐process based research.

**Figure 11:**
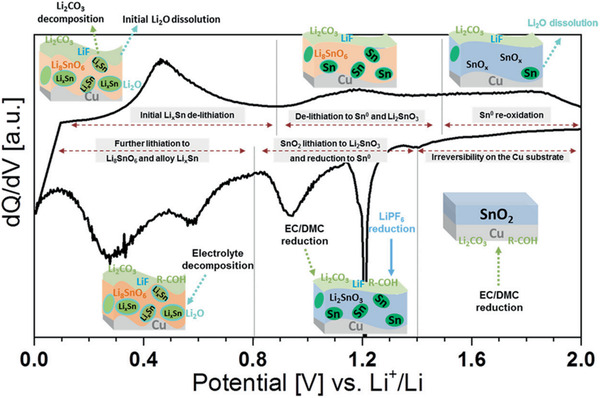
Reactions and phase formation occurring at different potentials versus Li/Li^+^ during lithiation/delithiation of a SnO_2_ thin‐film electrode. Reproduced with permission.^[^ ^185^ ^]^ Copyright 2018, American Chemical Society.

SnO_2_ is among the most attractive conversion electrodes due to its high theoretical capacity of 1491 mAh g^−1^ provided by the incorporation of up to 8.4 Li‐ions per formula unit in sequential conversion and alloying processes. A review about SnO_2_‐based conversion anodes was published by Zoller et al.^[^ ^182^ ^]^ Unfortunately, thin films show rapid capacity fade when cycled with liquid electrolytes.^[^ ^183^ ^]^ For SnO_2_ deposited by PLD, a discharge capacity of 1426 mAh g^−1^ was reached during the first cycle, whereas within 50 cycles the capacity was decreased to 564 mAh g^−1^.^[^ ^184^ ^]^ Using thin amorphous SnO_2_ films deposited as a model electrode by d.c. magnetron sputtering, Ferraresi et al. were able to identify various intermediate conversion and alloy reactions taking place during lithiation/delithiation of tin oxide, and elucidated their impact on the total reversibility of the process.^[^ ^185^ ^]^ XPS and SEM analysis at several charged and discharged states, corroborated by density functional theory calculations, revealed that Li_2_SnO_3_ and Li_8_SnO_6_ were formed as intermediate phases during the lithiation process (**Figure** **11**).^[^ ^185^ ^]^

A thin film battery consisting of a LMO cathode, a LiPON electrolyte and an SnO_2_ anode showed a strong capacity loss of about 60% after 50 cycles, which was attributed to the poor performance of the SnO_2_ anode.^[^ ^113^ ^]^ PLD of a nanocomposite consisting of alternating amorphous SnO_2_ with 60 nm thickness and TiO_2_ with 10 nm thickness afforded a capacity of about 160 μAh cm^−2^ for a 210 nm thick composite cycled with liquid electrolyte for 200 cycles.^[^ ^183^ ^]^ A pure SnO_2_ thin film with the same thickness showed severe cracking after cycling, whereas the multilayer structure remained intact. The increased capacity retention of the multilayer system was attributed to the mechanical constraint of the SnO_2_ layers.^[^ ^183^ ^]^

Nitrides are particularly attractive as conversion‐type electrodes, because ionically conductive Li_3_N with a Li‐ion conductivity of up to 1.2 × 10^−3^ S cm^−1[^ ^186^ ^]^ is formed as a reaction product. This formation establishes a Li‐ion conduction path inside the anode, in contrast to insulating Li_2_O formed during conversion of oxide materials. SnN*_x_* thin films deposited by a reactive sputtering process yielded capacities of up to 700 μAh cm^−2^ µm^−1^ for the compositions Sn:N = 1:1 and Sn:N = 3:4 in cells with liquid electrolyte.^[^ ^187^ ^]^ Long‐term cycling showed a better performance in case of the 1:1‐composition, although a strong degradation within the first 100 cycles was also observed. It was shown that the reduction of the cut‐off potential from 2 to 0.8 V reduces the capacity of the SnN film to about 80% of its initial value but significantly improves the cycling stability. Even the thickest film, which rapidly delaminated when cycled to its full capacity, could be cycled for 120 cycles without significant capacity loss in the reduced potential range.^[^ ^187^ ^]^

**Figure 12:**
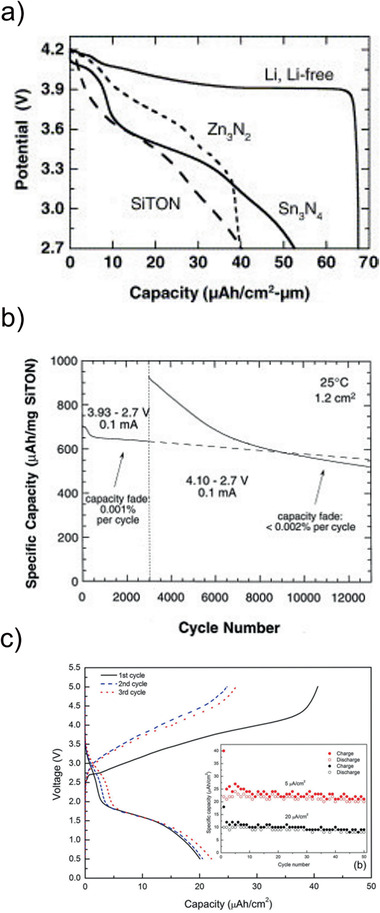
Cycling properties of thin‐film all‐solid‐state batteries with conversion‐type thin‐film anode. a) Discharge curve of SiTON, Zn_3_N_4_ and Sn_3_N_4_ conversion anode in comparison to in‐situ plated Li‐anode. Reproduced with permission.^[^ ^188^ ^]^ Copyright 2005, Elsevier b) long‐term cycling of a SiTON/LiPON/LCO thin‐film battery. Reproduced with permission.^[^ ^191^ ^]^ Copyright 1999, Elsevier. c) Charge–discharge curve at low current density and cycling performance at high and low current density of a ZnO/LiPON/LMO thin‐film battery. Reproduced with permission.^[^ ^196^ ^]^ Copyright 2018, Elsevier.

By employing sputtering, Dudney^[^ ^188^ ^]^ and Neudecker et al.^[^ ^189^ ^]^ have provided the first evidence that Sn_3_N_4_ layers can be used as anodes in all‐solid‐state cells. However, the discharge capacities of the cells built with a LiPON electrolyte, a LCO cathode and a Sn_3_N_4_ anode were still inferior to similar “anode‐free” cells or cells with a Li‐anode (**Figure** **12**a).^[^ ^188^, ^189^ ^]^ A Sn_3_N_4_ anode was shown to be superior to comparable Zn_3_N_2_, Sn_3_S_4_ and InN*_x_* anodes.^[^ ^188^, ^189^ ^]^ In another example published by Li et al., a LCO/LiPON/SnN*_x_* cell fabricated using various sputtering processes showed a maximum discharge capacity at 60 °C, whereas the discharge capacity decreased upon further increasing the temperature. Constant discharge capacities were observed during cycling at 20 and 100 °C for 15 cycles.^[^ ^190^ ^]^

Silicon tin oxynitride (SiTON) is another conversion material with promising performance characteristics as a negative electrode in solid‐state batteries. Amorphous SiTON films can be deposited by r.f. sputtering from a SnSiO_3_ target in a nitrogen plasma.^[^ ^191^ ^]^ Full LCO/LiPON/SiTON cells showed a discharge capacity of 340 mAh g^−1^ (with respect to the anode) without annealing. Exposure of the cell to 250 °C (the temperature required for soldering targeted as a possible application for the cell) resulted in an improved discharge capacity of up to 450 mAh g^−1^. The cells showed a low capacity fade of 0.001% per cycle when cycled between 2.7 and 3.93 V and 0.002% per cycle when cycled between 2.7 and 4.1 V (Figure 12b). The latter condition detrimentally affected the cycling stability but increased the discharge capacity by almost 50 mAh g^−1^.

Besides tin‐based materials, titania‐based compounds attracted attention as conversion‐type electrode materials. TiO_2_ anodes can be easily obtained by a reactive d.c. sputtering process with oxygen. Generally, the crystal structure of sputtered TiO_2_ thin films can be adjusted by controlling the oxygen partial pressure and total pressure during deposition.^[^ ^192^ ^]^ However, the maximum capacity obtained in liquid electrolyte cells was only 330 mAh g^−1^ at 0.2 C,^[^ ^192^ ^]^ which is relatively low in comparison to other anode materials. A 200 nm thick amorphous TiO_2_ film was used as an anode in a battery based on a 250 nm thick NMC111 cathode and a LiPON electrolyte.^[^ ^193^ ^]^ This cell showed an initial discharge capacity of 52 μAh cm^−2^ µm^−1^, and still exhibited 86% of the initial capacity after 100 cycles. The cell showed high polarization due to the absence of conductive additives in the electrodes. Therefore, cycling was carried out between 3 and 5 V. The high upper cut‐off voltage can lead to degradation of the cathode, which could explain the low capacity as well as the deterioration of the cell.^[^ ^193^ ^]^

ZnO is another interesting type of anode material in which a conversion reaction to Zn and Li_2_O is followed by alloying of Zn and Li, which theoretically enables the accommodation of up to 3 Li ions per unit structure. Studies on thin ZnO films deposited by PLD and sputtering from ceramic targets showed, however, that a large amount of capacity, e.g., 55% or 44% was lost during the first charge cycle for undoped ZnO layers.^[^ ^194^, ^195^ ^]^ Doping with Al is an efficient means to improve the cycling performance of such sputtered thin films. Doping up to 3 wt% Al increased the cycling stability within the first 40 cycles significantly, so that capacities of about 500 mAh g^−1^ could be obtained.^[^ ^195^ ^]^ However, higher amounts of Al dopants have a detrimental effect on the electrode performance. The electronic conductivity was improved by Al doping up to 3 wt%, as was confirmed by Hall measurements. The introduced Al is converted into electrochemically inert nanocrystalline Al_2_O_3_ after the first lithiation and delithiation process. It was assumed that these nanocrystals influence the cycling behavior of ZnO directly, most likely through their function as a stress releaser.^[^ ^195^ ^]^ Aside from investigations on individual layers, ZnO was also tested as a negative electrode in full solid‐state cells. A thin film battery based on an amorphous LMO cathode, a LiPON electrolyte and a ZnO anode showed a low discharge capacity of 22 μAh cm^−2^, which was mainly attributed to the amorphous nature of the cathode.^[^ ^196^ ^]^ A beneficial effect of using ZnO instead of metallic Li anodes is the stability of cells in air. Thus the cells protected by a LiPON layer without any additional sealing were cycled 50 times in air without significant capacity loss (Figure 12c).^[^ ^196^ ^]^

## 4 Interfaces—Model Systems and Modifications

Interfacial processes play a central role in any electrochemical energy storage system. Therefore, understanding and optimization of interfaces between different battery materials have always been the focus of PVD research. Due to the possibility of producing thin layers with a defined thickness, chemical composition, phase composition, and crystallinity as well the option of sequentially depositing multiple layers, various PVD processes are ideally suited to fabricate model systems to study interfacial phenomena. Comparatively low process temperatures in PVD methods are often of advantage for obtaining clean defined interfaces between solid materials that would otherwise require high temperature sintering with possible side reactions. Further, the layered geometry of the obtained systems is well suited for the characterization of the structure and composition with advanced analytics (e.g., XPS, SIMS and SEM) in combination with electroanalytical techniques, providing deeper insights into the structure as well as charge transfer and transport properties of various interfaces. Besides fundamental studies of interfacial properties, PVD is frequently applied to produce thin functional layers to improve the properties of the interfaces, such as protective coatings to prevent chemical or electrochemical reactions between battery materials during processing or operation, or coatings to improve the contact between the materials and minimize the interfacial resistance.

This section provides an overview of examples of PVD processes applied for the investigation and optimization of interfaces between active electrode materials and electrolytes.

### 4.1 Interfaces between Cathodes and Solid Electrolytes

The high impedance at the interface between the CAM and the electrolyte is one of the key reasons for the high total impedance and rapid deterioration of the complete battery. The high interfacial resistance can be caused a) by the processing conditions (e.g., formation of high impedance reaction products during heating), which is often the case for solid state materials, b) due to electrochemical reactions during operation or c) contact loss due to electrochemo‐mechanical processes at the interface (such as volume changes) during operation.

One concept to describe the interfacial impedance at the electrode/electrolyte interface is the formation of a space charge layer. In this scenario a potential difference between the electrode and the electrolyte causes Li‐ion migration from the electrolyte to the CAM and from the anode to the electrolyte resulting in a space charge layer at the interface. The impact of the space charge layer on the overall cell performance is still a subject of controversial discussion. For instance, the group of Wagemaker and coworkers claimed that the space charge layer thickness in solid electrolytes is too small (≈1 nm) to be of any significance.^[^ ^197^ ^]^

LiPON is one of the most important solid electrolytes used in commercial all‐solid‐state batteries (see Section 5.1.1). Therefore, the processes at the interface of LiPON with different CAMs have been intensively investigated using PVD methods. LiPON is practically exclusively fabricated by PVD processes at temperatures below 200 °C. Hence one can expect that processing‐induced detrimental reactions at the interface (material interdiffusion) are negligible and do not contribute to the interfacial resistance. However, for the interface between LiPON and LCO a high interface resistance of 300 Ω cm^2^ was detected by impedance spectroscopy.^[^ ^198^ ^]^ This resistance could be reduced to 125 Ω cm^2^ by heat treatment at 200 °C for 60 min in air.^[^ ^199^ ^]^ As changes in the activation energy of the charge transfer process were not detected, the structural changes at the interface during heat‐treatment were hypothesized as the main reason for the reduced resistance.^[^ ^198^ ^]^ Gittleson and El Gabaly reported that the cycling stability of the LCO/LiPON interface can be slightly improved by a PLD deposition of an intermediate LiNbO_3_ layer.^[^ ^200^ ^]^

**Figure 13:**
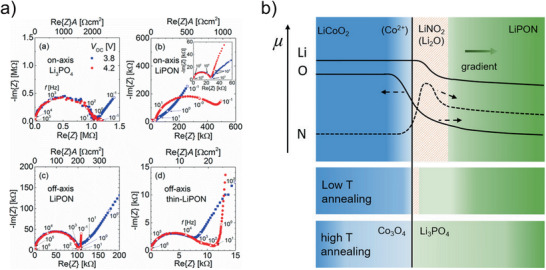
a) Impedance spectra of thin‐film batteries with Li_3_PO_4_ or LiPON electrolytes deposited under different deposition conditions: On‐axis in Ar atmosphere (upper left), on‐axis in N_2_‐atmosphere (upper right), 1500 nm thin film in off‐axis configuration (lower left) and 100 nm in off‐axis configuration (lower right). Reproduced with permission.^[^ ^201^ ^]^ Copyright 2015, American Chemical Society. b) Schematic illustration of LiPON‐LiCoO_2_ interface—after deposition (upper part) and after annealing (lower part). Reproduced with permission.^[^ ^203^ ^]^ Copyright 2017, American Chemical Society.

Haruta et al. reported a significantly reduced resistance at the LCO/LiPON interface as a result of off‐axis sputtering (**Figure** **13**a).^[^ ^201^ ^]^ The deterioration of the interface during their on‐axis sputtering process led to an interface resistance of 880 Ω cm^2^. It was assumed that the interface damages are caused by a bombardment of the interface with N^−^‐ and O^−^‐ions.^[^ ^201^ ^]^ Sputtering without nitrogen led to a reduced resistance of 200 Ω cm^2^ for the interface between amorphous Li_3_PO_4_ and LCO.^[^ ^201^ ^]^ Lower values of 90 Ω cm^2^ were reported for a Li_3_PO_4_/LCO interface, where both layers were deposited by PLD.^[^ ^42^ ^]^ The off‐axis sputtering approach reduced the resistance at the LCO/LiPON interface to 8.6 Ω cm^2^. The authors concluded that a space charge layer was not present at the LCO/LiPON interface and that a major part of the resistance was caused by defects produced by the sputtering process.^[^ ^201^ ^]^

The formation of the processing‐induced reaction layer on the LCO/LiPON interface as a possible reason for the high interfacial resistance was confirmed by Jacke et al.^[^ ^202^ ^]^ XPS studies demonstrated that during the deposition a very thin layer of about 10 Å was formed. It consisted of nitrogen‐and oxygen containing species, such as NO_2_^−^ and NO_3_^−^.^[^ ^202^ ^]^ Detailed measurements during annealing revealed a complete loss of these species at temperatures above 200 °C and a reaction between LCO and LiPON at temperatures higher than 350 °C, which resulted in the formation of Co_3_O_4_, Li_3_PO_4_, and structurally altered LiPON (Figure 13b).^[^ ^203^ ^]^

Recently, a low resistance of 10.2 Ω cm^2^ was also reported for the LiNi_1/3_Mn_1/3_Co_1/3_O_2_ (NMC111)/Li_3_PO_4_ interface.^[^ ^103^ ^]^

Aside from LCO, spinel structured electrodes were studied as cathode materials in combination with LiPON, and the interfacial properties of such systems were investigated. As deposited LMO/LiPON/Li cells showed poor cycling behavior,^[^ ^112^ ^]^ but after annealing at 498 K the cathode performance was similar to that obtained with a liquid electrolyte cell. Despite the greatly improved cell performance, the LMO/LiPON interface showed a high resistance of 2500 Ω cm^2^ during the first charging cycle at a potential of 4 V. The resistance further increased to 15 000 Ω cm^2^ after the 500^th^ cycle.^[^ ^112^ ^]^ A much lower resistance was observed for the interface between LiPON and a LNMO cathode layer. For the respective LNMO/LiPON/Li thin‐film battery a total interface area specific impedance of 203 Ω cm^2^ was reported, which slightly increased to 229 Ω cm^2^ after 4000 cycles.

The charge‐transfer resistance at the LiPON‐LiCr_0.05_Ni_0.45_Mn_1.5_O_4_ interface was lowered by the deposition of BaTiO_3_ (BTO) nanoparticles.^[^ ^204^ ^]^ The particles were applied by electrospray deposition of a suspension with mass loadings of up to 8 wt%. The best electrochemical performance in comparison to the unmodified interface was observed with a mass loading of 0.008%. The discharge capacity was increased from 45 to 120 mAh g^−1^ at a rate of 0.25 C for the first cycle. It was assumed that in close proximity to the dielectric BTO nanoparticles the charge difference is compensated, and thus a pathway for Li‐ions is generated at the interface.^[^ ^204^ ^]^

The significant differences in the reported spinel‐LiPON interface resistances could also be attributed to a possible deterioration of the interface during LiPON sputtering as it was reported for LCO thin films. This hypothesis is supported by the low interface resistances that were reported for LNMO/Li_3_PO_4_ deposited by PLD, namely 7.6 Ω cm^2^ for (100) LNMO^[^ ^205^ ^]^ and 34 Ω cm^2^ for (111) LNMO.^[^ ^206^ ^]^

For multilayered systems, the PVD deposition sequence, the processing temperature, and possible interactions between the materials during deposition and heating have a significant effect on the properties of the resulting interfaces. The latter is particularly relevant for crystalline electrolytes other than LiPON, which usually require relatively high deposition and post‐annealing temperatures to ensure their crystallization. To minimize the reaction between the materials, the layer that crystallizes at the lower temperature (typically the CAM) can be deposited on top of the crystalline ceramic layer (typically the solid electrolyte), which is often used for the fabrication of ceramic model systems. However, even in this case, the detrimental reactions at the interface cannot be suppressed completely. Therefore, the interfacial resistance between the CAM and crystalline ceramic electrolytes is always higher than that of similar interfaces with LiPON.

For layered CAM/ceramic electrolyte interfaces the corresponding resistance strongly depends on the CAM deposition conditions, and it typically increases after annealing. Experimental data showed that the resistance of the LCO/LATP interface was 400 Ω cm^2^ after deposition, but it increased to 9750 Ω cm^2^ after annealing at 500 °C (measured at 50 °C).^[^ ^207^ ^]^ Similar results were observed for LiMn_0.5_Ni_0.5_O_2_^[^ ^208^ ^]^ and NMC111^[^ ^209^ ^]^ deposited on top of crystalline LATP layers. The interface resistance increased strongly with increasing annealing temperature, for example, a value of about 1000 Ω cm^2^ at 50 °C was determined for the LiMn_0.5_Ni_0.5_O_2_ interface after annealing at 300 °C.^[^ ^208^ ^]^ The initial discharge capacity of this system was low (<100 mAh g^−1^), which was attributed to insufficient cation ordering caused by the low annealing temperature.^[^ ^208^ ^]^ Similar cells with as‐deposited LCO^[^ ^207^ ^]^ and NMC111^[^ ^210^ ^]^ thin films showed higher initial capacities. However, long‐term cycling data was not presented.

**Figure 14:**
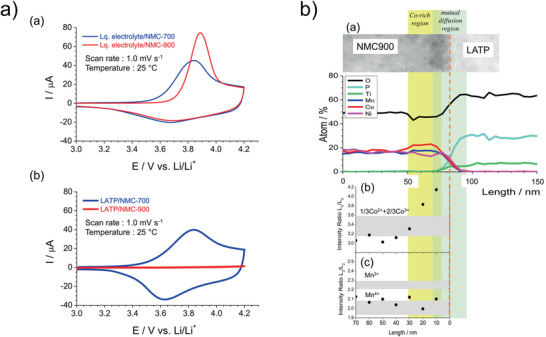
a) Cyclic voltammograms of NMC111 deposited at 520 °C (labeled as NMC700) and 670 °C (labeled as NMC900) on Pt‐coated SiO_2_ cycled with a liquid electrolyte and a Li anode (top) and the same NMC111 deposited on LATP ceramic and cycled in polymer/ceramic hybrid cell(bottom) with a Li anode. b) Chemical analysis of NMC111 deposited at 670 °C on LATP shows a Co‐rich layer close to the interface. Reproduced with permission.^[^ ^209^ ^]^ Copyright 2016, Elsevier.

LCO deposited at a substrate temperature of 600 °C on LATP led to an interface resistance of about 5900 Ω cm^2^.^[^ ^211^ ^]^ NMC111 deposited by PLD showed interface resistances of 315 Ω cm^2^ and 114 000 Ω cm^2^ for samples deposited at surface temperatures of 520 and 670 °C, respectively.^[^ ^209^ ^]^ Further, no electrochemical activity was observed for the sample deposited at 670 °C (**Figure** **14**a). The high resistance values for the samples deposited at elevated temperatures can be attributed to the reactions between the NaSICON materials and oxide cathodes, which was detected for bulk materials at temperatures above 600 °C.^[^ ^212^ ^]^ This hypothesis was supported by results obtained by Raman^[^ ^207^ ^]^ and X‐ray absorption spectroscopy,^[^ ^211^ ^]^ which indicated the formation of Co_3_O_4_ at the LCO/LATP interface. Rutherford‐backscattering spectroscopy also revealed the presence of a reaction layer at the cathode/electrolyte interface of post‐annealed samples.^[^ ^213^ ^]^

Similar results were also obtained with a NMC/LATP sample deposited at 670 °C, which showed the presence of a Co‐rich layer at the interface (Figure 14b).^[^ ^209^ ^]^ It is assumed that Co^2+^, which is formed as a consequence of oxygen release from the NMC (this reaction is possibly facilitated in the presence of LATP), diffuses to the interface and forms highly resistive phases, for example, MnCo_2_O_4_, which were detected by XRD.^[^ ^209^ ^]^

The reaction between the materials can be mitigated to some degree by the application of a buffer layer. For the LCO/NaSICON system, the introduction of a niobium oxide layer between LCO and NaSICON led to a significant reduction of the interface resistance to less than 2000 Ω cm^2^ as compared to 5900 Ω cm^2^ for the bare interface.^[^ ^211^ ^]^ The activation energy of the Li‐ion transfer at the interface was also reduced. Other coatings, such as zirconium oxide, did not cause any significant changes regarding the interface resistance, while the deposition of a molybdenum oxide buffer layer even increased the interface resistance in comparison to the uncoated sample.^[^ ^211^ ^]^

Similar to NaSICON, garnet‐based solid electrolytes also undergo reactions with all state‐of‐art cathode materials far below their sintering temperature (about 1100–1200 °C). The reactions between LLZ and different CAMs take place at temperatures between 400 and 700 °C.^[^ ^58^, ^214^, ^215^ ^]^. Only LCO was reported to be relatively stable at elevated temperatures.^[^ ^59^, ^215^ ^]^ However, the interdiffusion of Al from Al‐substituted LLZ into LCO was observed at 700 °C accompanied by the formation of tetragonal LLZ, which has a lower ionic conductivity than cubic LLZ.^[^ ^216^ ^]^

**Figure 15:**
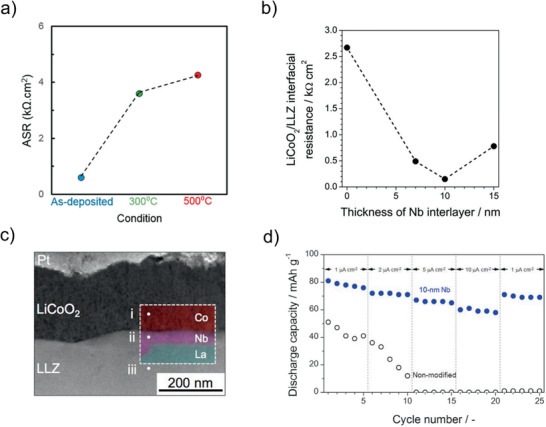
a) Interface resistance of post‐annealed LCO thin film on LLZ ceramic pellet. Reproduced with permission.^[^ ^217^ ^]^ Copyright 2018, American Chemical Society. b) Interface resistance of hot‐deposited LCO on LLZ pellet with Nb‐interlayer as a function of layer thickness. Reproduced with permission.^[^ ^219^ ^]^ Copyright 2014, Elsevier. c) SEM image of LCO/LLZ interface with Nb interlayer. Reproduced with permission.^[^ ^219^ ^]^ Copyright 2014, Elsevier. d) Cycling properties of LCO/LLZ/Li cell with and without Nb interlayer (LCO/Nb/LLZ/Li). Reproduced with permission.^[^ ^219^ ^]^ Copyright 2014, Elsevier.

Sputter deposition of LCO on Al‐doped LLZ with subsequent post‐annealing between 300 and 500 °C resulted in the formation of an interface layer with a thickness of about 100 nm already at these relatively low processing temperatures.^[^ ^217^ ^]^ The interface resistance increased from 600 to 4300 Ω cm^2^ for the as‐deposited sample and the sample annealed at 500 °C, respectively (**Figure** **15**a).^[^ ^217^ ^]^

Nevertheless, a model battery with a 500 nm thick LCO cathode that was annealed at 600 °C after the PLD process did not show significant degradation during 100 charge–discharge cycles.^[^ ^218^ ^]^ The discharge capacity for the first cycle and the 100th cycle was 129 and 127 mAh g^−1^, respectively.

PLD of LCO on LLZ ceramics carried out at 600 °C led to slightly lower interface resistance of about 2600 Ω cm^2^.^[^ ^219^ ^]^ At a higher temperature of 700 °C the formation of La_2_CoO_4_ at the interface and a reduced electrochemical activity of the LCO electrode were observed.^[^ ^220^ ^]^ This reaction was not detected when the interface was modified by a very thin Nb‐O‐interlayer (thickness <15 nm), which was formed by sputtering of Nb metal and subsequent annealing at 600 °C in O_2_‐atmosphere (Figure 15b–d).^[^ ^219^ ^]^ A cell with a 10 nm thick Nb‐layer and an LCO cathode deposited at 600 °C by PLD showed a significantly reduced resistance of 150 Ω cm^2^. Battery cycling also showed lower degradation as well as improved capacity retention for the modified cell.^[^ ^219^ ^]^ A LiNbO_3_ interlayer was also successfully applied for the sputter deposition of LLZ on LCO.^[^ ^55^ ^]^ The diffusion of Co into the LLZ layer was suppressed. However, interdiffusion of Cr from the current collector was still evident from the SIMS depth profile.^[^ ^55^ ^]^

The performance and interfacial resistance of LLZ/LCO layers obtained by PVD are generally inferior to those of comparable bulk systems fabricated by classical ceramic approaches. A mixed LLZ/LCO composite obtained by co‐sintering of the respective powders demonstrated satisfactory electrochemical performance even after sintering at 1050 °C,^[^ ^221^ ^]^ whereas PVD bilayer electrodes severely degrade at these temperatures. The causes of these discrepancies in performance still have to be identified. Differences in the reaction kinetics of thin films as compared to crystalline powders, as well as differences in crystallinity and phase purity of PVD layers, and ceramic powders synthesized at high temperatures may be plausible explanations.

Although perovskite‐structured solid electrolytes have been known for almost 30 years, their interaction with CAMs has been investigated to a lesser degree compared to garnet‐ or NaSICON‐structured electrolytes. Similar to other material classes, the reactions between perovskite‐structured electrolytes and CAMs are observed at relatively low temperatures. Bulk reactions of Li_0.5_La_0.5_TiO_3_ with LCO and LiNiO_2_ powders were reported to occur above 700^[^ ^222^, ^223^ ^]^ and 500 °C,^[^ ^222^ ^]^ respectively. Li_0.5_La_0.5_TiO_3_ exhibited a much higher stability in combination with LMO at temperatures above 800 °C.^[^ ^222^, ^223^ ^]^

Interfacial and electrochemical properties of LCO/Li_0.5_La_0.5_TiO_3_ electrodes obtained by PLD at 700 °C are strongly affected by the treatment of the Li_0.5_La_0.5_TiO_3_ surface.^[^ ^224^, ^225^ ^]^ In cyclic voltammetry studies, the peak separation of the Co^3+/4+^ redox couple was 0.35 V during the first cycle for a cleaved LLT surface, and it further increased with continuous cycling.^[^ ^225^ ^]^ In contrast, a polished sample showed a peak separation of only 0.18 V and no increase with an increasing number of cycles.^[^ ^225^ ^]^ An even smaller peak separation was observed for an Ar‐ion polished sample.^[^ ^224^ ^]^ TEM‐analysis revealed an intergrowth of the cleaved (110) Li_0.5_La_0.5_TiO_3_ plane with the (11‐20) plane of LCO, which is beneficial for Li‐ion conduction across the interface. However, during charge–discharge cycling, this plane undergoes a comparably large expansion and contraction, which causes mechanical stress at the interface resulting in a rapidly increased cell resistance.^[^ ^225^ ^]^ In contrast, the polished and the Ar‐ion treated surface exhibited an amorphous layer at the interface, which led to a random orientation of the LCO grains at the interface and therefore reduced mechanical stress during cycling.^[^ ^224^, ^225^ ^]^ It was assumed that the beneficial Li‐ion conducting properties of amorphous LLT improved the Li‐ion transfer at the interface.^[^ ^224^, ^225^ ^]^

The results shown in this section demonstrate that side reactions can occur at the interface in thin film processes. Therefore, the chemical and electrochemical properties of a system should always be considered together in order to separate side reactions from actual interfacial phenomena. Nevertheless, the low interface resistances between Li_3_PO_4_‐based glassy electrolytes and the CAM show that with appropriate processing the interfacial resistances in solid‐state batteries can closely match those observed for state‐of‐the‐art batteries with liquid electrolyte.

### 4.2 Interfaces between Anodes and Solid Electrolytes

The utilization of lithium metal anodes is one of the “holy grails” in battery development. Therefore, the investigation of the interface between the lithium anode and the electrolyte is one of the most important aspects in battery research. LiPON is one of the few electrolytes that show stable cycling behavior in contact with Li metal. However, XPS investigations of the Li/LiPON interface demonstrated that LiPON is not chemically stable in contact with Li. Reactions at the interface lead to the formation of Li_3_PO_4_, Li_3_N, and Li_2_O, which are ionically conductive and apparently form a solid electrolyte interface, which protects LiPON from further degradation.^[^ ^226^ ^]^

Besides Li foil anodes, so‐called “Li‐free” cells have attracted increasing attention as a potential cell concept. Li‐free cells do not contain Li metal initially. The anode layer is formed during the first charge cycle via electroplating at the interface of the electrolyte and the current collector.^[^ ^227^ ^]^ The concept of Li‐free cells is appealing because of their potentially lower production costs and improved safety. However, the cell performance strongly depends on the electroplating kinetics, the stability of anode interfaces and the possible degradation of formed thin Li layers due to reactions with battery components and the atmosphere if the cell is not sealed. The impact of cell sealing on the Li degradation was investigated by Neudecker et al. with a Cu current collector that was deposited on LiPON/LCO cells via sputtering. The batteries without an airtight barrier showed strong degradation during the first cycle due to the immediate irreversible reaction of electroplated Li with residual oxygen and moisture in the glovebox on the surface of the current collector. In contrast, Li‐free cells covered with LiPON or parylene C showed electrochemical properties that were similar to Li‐cells. A detailed in situ microscopy study of the Li‐plating mechanism at the interface between LiPON and a Cu current collector foil was carried out by Sagane et al. The size of Li precipitates decreased with increasing current density.^[^ ^228^ ^]^ The concept of Li‐free batteries was also investigated with an inverted battery structure, which means the sequence of the cell components was switched from substrate‐cathode‐electrolyte‐anode to substrate‐(anode)‐electrolyte‐cathode.^[^ ^229^ ^]^ In this case, LiPON with a thickness of 30 nm was directly deposited on a platinum coated stainless steel current collector by r.f. sputtering, and a crystalline LCO cathode was deposited on top of LiPON by aerosol deposition. The cell showed an initial discharge capacity of around 110 mAh g^−1^, and after 100 cycles the capacity was still at 90 mAh g^−1^.^[^ ^229^ ^]^ In contrast to the Li free battery shown by Neudecker et al.,^[^ ^227^ ^]^ sealing was not necessary to protect the Li‐anode, because it appeared to be completely covered by the solid electrolyte.

Using in situ SEM, Santhanagopalan et al. showed the appearance of a Si—P‐intermixing layer at the Si‐LiPON interface with simultaneous Li‐plating at the Si—current collector interface during overcharging of a Si/LiPON/LCO thin‐film battery.^[^ ^230^ ^]^

Besides LiPON, garnets showed good stability in contact with Li‐metal, as was indicated by ab‐initio calculations and verified by in situ XPS measurements.^[^ ^231^ ^]^ However, the formation of dendrites in garnet electrolytes is a significant issue to be addressed.^[^ ^232^ ^]^ Insufficient contact between the Li‐metal anode and the garnet electrolyte and therefore high interfacial resistances of about 1 kΩ cm^2^ were suspected to cause the dendrite growth.^[^ ^156^, ^233^, ^234^, ^235^ ^]^ By deposition of buffer layers, such as Au^[^ ^233^ ^]^ (deposited by d.c. sputtering), ZnO^[^ ^234^ ^]^ (deposited by atomic layer deposition (ALD)), Al_2_O_3_^[^ ^235^ ^]^ (deposited by ALD) or Ge^[^ ^156^ ^]^ (deposited by evaporation), the interface resistance could be lowered to 380, 20, 176, and 115 Ω cm^2^, respectively. Nevertheless, an increase of the critical current density above 0.2 mA cm^−2^ was not possible for planar electrodes. Further, it was shown that the interface resistance was mainly caused by impurities on the surface due to the facile reaction of LLZ with moisture. Proper surface cleaning combined with optimized heat treatment also led to low interface resistances of about 2 Ω cm^2^.^[^ ^236^ ^]^ Currently, the general application of Li‐metal anodes is investigated.^[^ ^237^ ^]^ 3D structuring of the anode^[^ ^238^ ^]^ and alternative materials seem to be more advanced solutions.^[^ ^239^ ^]^ Although the dendrite formation of Li‐metal anodes was studied in particular on garnet‐structured Li‐ion conductors, it should be mentioned that the same issues have to be addressed for other solid electrolytes.

The interface between crystalline LLZ pellets and Sn and Si alloy anodes deposited via PVD was investigated by Ferraresi et al.^[^ ^240^, ^241^ ^]^ The Si thin‐film anode showed a high initial capacity, but the capacity dropped significantly after few cycles. Within the first 20 cycles the capacity dropped from 2700 mAh g^‐^^1^ to 2000 mAh g^−1^. Further, cycling at different C‐rates demonstrated that the lithiation process was kinetically limited, whereas the delithiation process was not affected.^[^ ^240^ ^]^ The Sn model anode had a low initial capacity, which increased during cycling, most likely due to improved contact. Lithiation of the Sn‐anode by application of Li‐metal above the Li melting temperature resulted in an anode with stable cycling behavior and a reduced interface resistance.^[^ ^241^ ^]^

NaSICON based electrolytes are not stable in contact with Li‐metal due to their narrow electrochemical window, which lies in the range between 2.17 V and 4.21 V and between 2.70 V and 4.27 V for LATP and for LAGP,^[^ ^242^ ^]^ respectively. After contact with Li‐metal, the reduction of Ge^4+^ and Ti^4+^ to lower oxidation states was detected by sputter‐XPS.^[^ ^243^ ^]^ A decrease of the total Li‐ion conductivity of about one order of magnitude and an increase of the electronic conductivity of about three orders of magnitude were observed as a result of the interface degradation.^[^ ^243^ ^]^

To prevent the reaction with Li, various protection layers were deposited via PVD on top of NaSICON electrolytes. The use of LiPON as a protective layer is obvious, and therefore it was deposited on NaSICON ceramics, enabling the application of Li without electrochemical reduction at the interface. In spite of the much lower conductivity of LiPON as compared to NaSICON, the total conductivity of electrolyte separator was not significantly affected. Hence, the coated separators with a thickness of about 470 µm still showed a high ionic conductivity of 1 × 10^−4^ S cm^−1^.^[^ ^244^ ^]^ Detailed studies by XPS on the deposition of LiPON on 150 µm thick NaSICON sheets revealed a reduction of titanium species at the surface due to the nitrogen plasma and the formation of Li_2_TiO_3_ at the interface at the beginning of the deposition process.^[^ ^245^ ^]^ However, this reaction was not observed after a certain LiPON thickness was reached due to the protective function of LiPON. Impedance analysis showed an increase of the surface resistance of NaSICON after nitrogen plasma treatment, even without LiPON deposition. Further, the lower conductivity of the 200 nm thick LiPON deposited on each side of the NaSICON sheet led to a significantly reduced overall conductivity of the composite in comparison to the uncoated ceramic.^[^ ^245^ ^]^

While Li‐plating and stripping tests on symmetric Li/LAGP/Li cells showed a drastic increase in the interface resistance from about 7000 Ω cm^2^ to about 850 000 Ω cm^2^, the application of a 60 nm thick sputtered Ge interlayer reduced the interface resistance to about 400 Ω cm^2^ (as compared to 7000 Ω cm^2^) in the pristine state and 3200 Ω cm^2^ after 300 cycles of Li‐plating and stripping (compared to 850 000 Ω cm^2^ after 32 cycles).^[^ ^155^ ^]^

The application of a 200 nm thick ZnO layer by magnetron sputter deposition at the LATP/Li interface represents another approach to avoid detrimental reactions between lithium metal and NaSICON.^[^ ^246^ ^]^ During heating of the Li/ZnO/LATP half‐cell, a conversion reaction with formation of Zn and Li_2_O took place. This layer not only prevented the contact between Li and LATP but also reduced the interface resistance from about 91 000 Ω cm^2^ to about 400 Ω cm^2^. The interface resistance of the cells with interlayers was only slightly reduced to about 340 Ω cm^2^ after Li plating and stripping at 0.2 mA cm^2^, showing a stable interface, whereas the value for the uncoated sample increased to 214 000 Ω cm^2^ due to the interface instability.^[^ ^246^ ^]^ Opposite results were obtained by Bai et al. who observed an increase in the interface resistance from 10 400 Ω cm^2^ to 365 000 Ω cm^2^ at 60 °C after coating LATP sheets with a 50 nm thick Al‐doped ZnO film. Long term cycling reduced the interface resistance slightly to about 345 000 Ω cm^2^.^[^ ^247^ ^]^ This strong deviation of results indicates that the processing conditions are crucial for the resulting interface resistance.

Perovskite electrolytes are not stable in contact with Li‐metal. In situ XPS measurements of LLT ceramics demonstrated the reduction of Ti^4+^ to Ti^3+^, Ti^2+^ and metallic titanium.^[^ ^248^ ^]^ Therefore, the perovskite electrolyte must be protected by an additional layer. Li et al. showed that LiPON can be efficiently used as a protective layer in a Li/LiPON/LLTO/LCO thin‐film cell, which was cycled for 100 cycles without a short‐circuit between 3 and 4.4 V.^[^ ^82^ ^]^

## 5 Commercial Thin Film Batteries and Development of New Cell Technologies

In this chapter the characteristics of mature full solid‐state cells as well as ongoing research topics with respect to full cells are discussed in view of current and potential applications. Some examples of commercially available batteries with thin‐film components are presented briefly. The realization of high voltage cells, which is one of the key benefits of replacing conventional liquid electrolytes with solid‐state components, is described as well as the development of flexible batteries for certain specialty applications. Further, efforts to improve the power and energy density through the development of 3D‐structured cells and the investigation of bulk cells are discussed.

### 5.1 Batteries with Thin Electrolyte Layers

Even though many types of solid electrolytes, including LiPON, exhibit relatively low bulk ionic conductivity, their application is still viable if the electrolyte layer is thin enough, and if the cell operates at relatively low rates. At present LiPON is the most commonly employed electrolyte material in commercial thin‐film solid‐state batteries, as is outlined in the following section. PVD fabrication processes allow the integration of the electrolyte and the active material under highly controlled conditions, affording very uniform components of the desired phase and composition.

#### 5.1.1 Commercial Thin Layer Batteries

Research focused on LiPON, which was conducted at Oak Ridge National Laboratory (ORNL) as described in Section 3.1.1, enabled the development and commercial deployment of solid‐state thin film lithium‐ion batteries. Examples of commercially available thin film batteries are presented in **Table** **1**. Infinite Power Solutions, Cymbet Corporation, and Front Edge Technology utilize ORNL's core technology, and each manufacturer offers several cell models with different capacities.

**Table 1:** Currently available commercial all‐solid‐state batteries

Company and model	Composition	Nominal capacity [mAh]	Thickness [µm]	Size [mm^2^]	Cycle life
Infinite Power Solutions, Inc. Thinergy MEC202^[^ ^249^ ^]^	LiCoO_2_/LiPON/Li	2.2	170	25.4 × 50.8	>10 000 cycles Industry standard with respect to lifetime
Cymbet Corporation EnerChip CBC050^[^ ^250^ ^]^	LiCoO_2_/LiPON/Li	0.05	200	5.7 × 6.1	>5000 cycles to 10% discharge
Front Edge Technology, Inc. NanoEnergy^[^ ^251^ ^]^	LiCoO_2_/LiPON/Li	5	400	42 × 25	<10% capacity loss over 1000 cycles when discharged at 1 mA cm^−12^
TDK Corporation CeraCharge 1812^[^ ^252^ ^]^	Electrolyte: Li_1.3_Al_0.3_Ti_1.7_(PO_4_)_3_, active material: Li_3_V_2_(PO_4_)_3_	0.1	1100	4.4 × 3.3	up to 1000

The cells are produced for a wide variety of applications, including mobile electronics, medical devices, and embedded systems, i.e., microbatteries that are integrated in electronic circuit boards. The cells are fabricated by sequential PVD steps. The industrial manufacturers provide specific barriers (enclosures) to protect the batteries from environmental influences, such as moisture from ambient air. Batteries from Infinite Power Solutions and Cymbet Corporation offer long cycle life. Cells from Cymbet Corporation operate reliably over 5000 cycles^[^ ^250^ ^]^ albeit not under deep discharge conditions, whereas Infinite Power Solutions’ technology exceeds 10 000 cycles at 100% depth of discharge. The latter corresponds to a battery life cycle of more than 15 years. Cymbet Corporation's batteries are designed for implementation in embedded systems. The capacity of their batteries can be either 5, 12, or 50 μAh. The charging time for the two cell types with higher capacity are 30 and 50 min.^[^ ^253^ ^]^ During storage at ambient conditions the self‐discharge rate is 2.5% per year. Charging to up to 90% of the available capacity is feasible within 15 min with Cymbet Corporation's cells.

Infinite Power Solutions specified a self‐discharge rate of <1% per year for their thin film battery products. For comparison, conventional LCO‐based batteries can lose 4% or more of their capacity when stored at 25 °C for one year, depending on the state of charge.^[^ ^254^ ^]^ Front Edge's NanoEnergy batteries, also based on ORNL's technology, are advertised as robust devices with respect to their charging behavior. A minimal temperature increase occurs during charging (<1 °C), and continuous charging at 4.2 V does not degrade the battery's performance. Since charging only occurs under constant voltage conditions, there is no danger of damaging the battery by overcharging it. This is one advantage compared to cells with liquid electrolyte in which overcharging causes electrolyte decomposition and concomitant capacity losses. The fully charged state is achieved within 4 and 20 min with the company's 0.25 and 0.9 mAh rated cells, respectively.^[^ ^251^ ^]^ The self‐discharge rate at ambient conditions is <5% per year.

A direct comparison of commercially available solid‐state thin‐film batteries with lithium metal anodes was conducted by Laïk et al.^[^ ^255^ ^]^ The names of the manufacturers or products were not disclosed by the authors. The key distinguishing features between the three cell types were the total thickness (80, 170, and 200 µm), the employed active material and the electrolyte. The two thicker cells contained a LCO cathode and a LiPON electrolyte, whereas the thinnest battery contained a transition metal oxide‐based cathode, which was not fully specified by the manufacturer, and its electrolyte component was described somewhat ambiguously as a conductive glassy inorganic thin film. Each cell type had a rated capacity of 0.7 mAh. However, the thickest cell delivered 0.9 mAh at 1 C. The thinnest battery exhibited the best performance overall. Its energy density of 42 mWh cm^−3^ was roughly twice as high compared to the other two models. At a rate of 1 C over 1000 cycles said cell featured full capacity retention at 100% depth of discharge, whereas the other two cells showed significant degradation. After about 200 cycles the 170 µm thick cell started to deteriorate, losing about 16% of its capacity over the full span of the test, while the thickest cell deteriorated steadily to the point of losing over 80% of its capacity in the range from the 500th to the 1000th cycle. Aside from the cell thickness, the choice of electrolyte in combination with the active material appeared to have played a pivotal role. To further investigate the degradation behavior, the capacity retention was assessed by comparing the performance at 20 °C before and after cycling at 60 °C. Initially, two cycles were carried out at 20 °C at the respective rates of C/50, C/5, 1 C, and 10 C. The subsequent cycling sequence at 60 °C consisted of 10 cycles at each of the same rates. Finally, a series of cycles, again at the same rates, was performed at 20 °C. The thin cell with the unspecified transition‐metal‐oxide based cathode and glassy phase electrolyte exhibited a high degree of capacity retention during this sequence of tests. In contrast, the other two cell types deteriorated, presumably due to decomposition effects at the interfaces between the electrolyte and the active material and at the current collector. At 1 C the two thicker cells each retained about 60% of the rated capacity, whereas the thin cell maintained about 97%.

In a recent report an experimental high performing thin‐film battery, which was also produced by a PVD process, was described. The battery featured high areal capacities of 0.89 and 0.45 mAh cm^−2^ at current densities of 10 µA cm^−2^ and 3 mA cm^−2^, respectively, and had areal dimensions of 3.1 mm × 1.7 mm and a thickness of 95 µm. It was composed of a 20 µm thick LCO cathode, a lithium‐free anode, a titanium current collector, and a LiPON electrolyte layer with a thickness of 3 µm.^[^ ^256^ ^]^ The overall strong performance characteristics were attributed to the high lithium diffusion coefficient of the lithium cobalt oxide cathode material (5 × 10^−9^ cm^2^ s^−1^). The ionic conductivity of the LiPON electrolyte was 3 × 10^−6^ S cm^−1^. The application for medical devices was explored as well by conducting electrochemical tests at 37 °C. At this elevated temperature, the available capacity increased by 15% compared to 25 °C. Further, a Ragone plot (energy density vs. power density) was generated, which showed increased energy densities and power densities compared to state‐of‐the‐art thin film battery technologies that are on the market. However, the cycling performance was less stable compared to commercial thin film batteries. Over 100 cycles the average capacity loss was 0.05% per cycle.^[^ ^257^ ^]^

The energy density of the thin‐film batteries presented in Table 1 is in the range of about 27 to 45 Wh l^−1^ (≈1–9 Wh kg^−1^). For comparison, commercial 18 650 type cells (i.e., Panasonic NCR18650PD) have an energy density of 590 Wh l^−1^ (235 Wh kg^−1^). The calendar life of such cells was estimated, indicating a predicted retention of the initial capacity of 84%–92% after 15 years at a temperature of 25 °C, the degradation being more pronounced when implementing a higher state of charge.^[^ ^258^ ^]^ The energy density of pouch cells manufactured by Contemporary Amperex Technology Co., Limited (CATL) was improved from 530 Wh l^−1^ (250 Wh kg^−1^) in 2017 to 700 Wh l^−1^ (300 Wh kg^−1^) in 2019, in part due to a modification of the cathode composition from NMC 532 to NMC 811.^[^ ^259^ ^]^

Generally, the potential to substantially increase the volumetric energy density and short charging times compared to incumbent lithium‐ion battery technologies with liquid electrolyte make solid‐state thin film batteries attractive for many fields. Further, the deposition techniques applied for the fabrication of thin film batteries are highly compatible with production processes for micro‐ and nanoscale devices. TDK developed a novel technology with their CeraCharge microbatteries, which contain purely ceramic electrode and electrolyte materials. The cells can function as surface mounted devices in electronics, and are applicable for energy harvesting, wearable electronics, medical devices and within the broad spectrum of what is commonly referred to as the Internet of things (IoT).^[^ ^260^ ^]^ The battery features a recurring multilayer structure composed of ceramic oxide films. The electrolyte consists of 97 wt% Li_1.3_Al_0.3_Ti_1.7_(PO_4_)_3_ and 3 wt% Li_3_PO_4_, and the electrodes are made of Li_3_V_2_(PO_4_)_3_, which functions as both the cathode and the anode active material. These components were not fabricated by PVD. The absence of flammable or highly reactive materials makes the battery inherently very safe. Based on the nominal voltage of 1.5 V, as specified by the manufacturer, the CeraCharge 1812 battery has an energy density of ≈9 Wh l^−1^ (3.8 Wh kg^−1^).

#### 5.1.2 Recent Developments in Thin Film Battery Research and Development

##### High‐Voltage Cells

The high electrochemical stability of LiPON, namely the ability to withstand voltages of up to 5.8 V,^[^ ^26^ ^]^ enables cycling of batteries with spinel‐structured high‐voltage cathode materials. This was demonstrated with a thin film battery based on a LNMO cathode, a LiPON electrolyte and a lithium anode, which exhibited stable cycling behavior for 10 000 cycles with discharge capacities of about 122 mAh g^−1^ and less than 0.001% capacity loss per cycle.^[^ ^261^ ^]^ Strong adhesion of the cell components and no indication of element interdiffusion were observed with a cell that was cycled for 1000 cycles. However, cycling at higher rates reduced the discharge capacity in comparison to a liquid electrolyte containing cell significantly. This behavior can be attributed to transport limitations of the LiPON electrolyte.^[^ ^261^ ^]^

**Figure 16:**
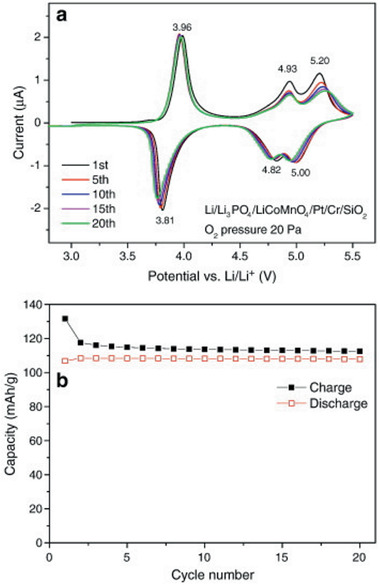
a) Cyclic voltammogram and b) cycling data of LCMO/LiPON/Li thin film cell. Reproduced with permission.^[^ ^116^ ^]^ Copyright 2014, Elsevier.

Batteries with discharge potentials above 5 V were realized by application of LCMO electrodes. Kuwata et al. fabricated a battery with a Li_3_PO_4_ electrolyte and a Li anode with a discharge capacity of 107 mAh g^−1^ and capacity retention of 99.4% between the 2nd and 20th cycle. The LCMO cathode was deposited at an oxygen pressure of 20 Pa (**Figure** **16**). Another sample deposited at an oxygen pressure of 100 Pa showed lower capacities (about 90 mAh g^−1^) and about 95% capacity retention between the 2nd and 20th cycle.^[^ ^116^ ^]^ In contrast, Li et al. cycled a battery consisting of a LCMO electrode, a LiPON electrolyte and a lithium anode in different voltage ranges. While during cycling with an upper cut‐off voltage of 5.5 V severe degradation over the first 20 cycles was observed, cycling to lower cut‐off voltages (1.4 V) showed improved cycling behavior when the upper cut‐off voltage was set to 5.0 V. The battery had a capacity of about 170.7 μAh cm^−2^ µm^−1^ (362.5 mAh g^−1^) with a capacity retention of 99.85% within the first 100 cycles in the voltage range between 1.4 and 5.0 V.^[^ ^122^ ^]^

##### Flexible Cells

**Figure 17:**
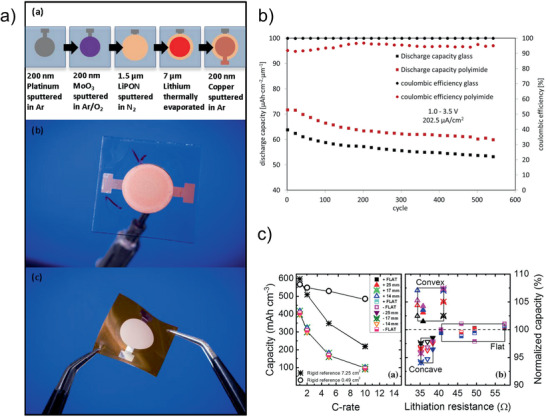
a) Setup and photograph of LiMoO_2_/LiPON/Li thin film cell on flexible polyimide substrate. Reproduced with permission.^[^ ^148^ ^]^ Copyright 2016, Elsevier. b) Cycling performance of LiMoO_2_/LiPON/Li thin film battery deposited on polyimide substrate in comparison to glass substrate. Reproduced with permission.^[^ ^148^ ^]^ Copyright 2016, Elsevier. c) Discharge capacity of bended cells in comparison to a rigid reference for a Li_4_Ti_5_O_12_/LiPON/Li battery on flexible YSZ substrate (left) Comparison of the different bending states in terms of normalized capacity and lithiation resistance (right). Reproduced under the terms of Creative Commons License CC BY 4.0.^[^ ^264^ ^]^ Copyright 2018, The Authors, published by Informa UK Limited.

Future applications, such as wearable electronics, might require flexible batteries. Several flexible cells have been realized on polymer substrates, for example, polyimide,^[^ ^148^, ^262^, ^263^ ^]^ and inorganic materials, such as thin and flexible YSZ sheets^[^ ^264^ ^]^ and mica layers.^[^ ^265^ ^]^ On polyimide substrates either amorphous cathodes, for example, MoO_3_ or V_2_O_5_, or LFP annealed at 400 °C were employed. For all cells LiPON was applied as the electrolyte and metallic lithium as the anode. A MoO_3_/LiPON/Li battery deposited on polyimide was cycled between 1 and 3.5 V and showed an initial discharge capacity of 180 μAh cm^−2^ µm^−1^ at 0.5 C, which decreased to 125 μAh cm^−2^ µm^−1^ during subsequent cycles. At a cycling rate of 10 C an initial capacity of 71.7 μAh cm^−2^ µm^−1^ was observed, and after 550 cycles 62.0 μAh cm^−2^ µm^−1^ still remained (**Figure** **17**a,b).^[^ ^148^ ^]^ Bending experiments performed on such cells showed a strong impact on the morphological properties. While the positive electrode delaminated under mild bending conditions, severe cracking in the electrolyte and anode layers was observed as a result of excessive bending.^[^ ^263^ ^]^ Koo et al. also investigated the electrochemical cell performance under bending conditions. The employed cell featured an LCO cathode, a LiPON electrolyte and a Li metal anode, which were sequentially deposited on a mica substrate. After heat treatment the mica layer was peeled off, and the cell was wrapped with polydimethylsiloxane (PDMS) sheets, so that the top and bottom of the cell were covered, and bending tests were performed thereafter. The total dimensions of this assembly were 2.54 cm x 2.54 cm x 0.2 cm while the cell itself occupied ≈50% of the footprint. Without bending the cell yielded an energy density of 2.2 × 10^3^ μWh cm^−3^ at a rate 46.5 µA cm^−2^ (corresponding to 0.5 C). Slight bending (radius = 16.0 mm) and harsher bending (radius = 3.1 mm) yielded initial capacities of 102 and 99 μAh cm^−2^, respectively, at said rate, while the capacity retention over 100 cycles was similar at ≈94–95% for both states.^[^ ^266^ ^]^

In contrast to polymer‐supported cells, batteries deposited on flexible YSZ substrates enable annealing of electrodes at elevated temperatures. Sepulveda et al. used YSZ as a substrate to build a flexible battery consisting of a LTO cathode (crystallized at 800 °C), a LiPON electrolyte and a Li anode.^[^ ^264^ ^]^ Bending had a strong influence on the achievable capacity. While concave bending led to a decrease of the discharge capacity by up to about 6%, the capacity was increased by convex bending by up to about 7% (Figure 17c).

##### 3D‐Structured Cells

At present, the areal energy density available from thin film planar microbatteries is still insufficient for many applications. This issue has prompted the drive toward realizing 3D microbatteries, which was initiated through work by Long and coworkers in 2004.^[^ ^267^ ^]^ In 3D batteries the total volumetric fraction of the inactive materials, for example, substrate and packaging, is reduced, which improves the energy density. Certain limitations regarding the transport kinetics associated with electrodes and interfaces can potentially be overcome with 3D‐structured batteries. Coating of 3D structures is conventionally carried out by CVD‐processes, which allow facile uniform coating of such relatively complex geometries.^[^ ^268^ ^]^ Pearse et al. investigated 2D and 3D structures produced by atomic layer deposition. Said structures consisted of a LiV_2_O_5_ cathode, a Li metal anode and a lithium polyphosphazene (LPZ, a polymorph of LiPON) electrolyte. The shift from a planar design toward a 3D structure, which increased the internal surface area tenfold relative to the planar surface of the 2D geometry, significantly improved the energy density and power density. The increase in the surface area resulted in an approximately tenfold increase of the power density reaching 1 mW cm^−2^.^[^ ^269^ ^]^

Processing cost and scalability are currently the main issues hindering the commercialization of 3D microbatteries.

The deposition of thicker electrode films with sufficient capacities is not economically viable when considering CVD processes for industrial applications due to the high cost of metal‐organic precursor materials. Therefore, the possibility of 3D batteries processed by PVD was also discussed in the literature. Xu et al. studied the coating behavior of LiPON as a function of the deposition angle on different 3D‐structured substrates.^[^ ^270^ ^]^ The group observed a decrease of the film thickness with increasing target distance and increasing incidence angle of the plasma. All films were uniform and exhibited a Li‐ion conductivity of about 2 ± 1 × 10^−6^ S cm^−1^. Therefore, the key requirements for coatings on 3D structures were fulfilled. Porous polycarbonate membranes, Ag epoxy posts and machined ridges within LCO‐structures were employed as 3D model systems. It became obvious that the deposition process must be adapted to achieve the required film thicknesses, because reduced deposition rates were detected locally inside deep and narrow structures.

The deposition of a full battery consisting of an LCO cathode, a LiPON electrolyte and a Si anode on a 3D‐structured silicon wafer yielded poor performance in comparison to a similar 2D battery.^[^ ^271^ ^]^ The main problem was the inhomogeneity of the deposited layers leading to a non‐uniform distribution of the current density, which was corroborated by a FEM model.

Instead of preparing a substrate with a tailored structure, the desired pattern can also be realized directly on the electrode. This can be achieved by the direct structural modification of an electrode surface through laser evaporation of the electrode material.^[^ ^272^ ^]^ 3D structuring can also be obtained by changing the growth mechanism of the cathode thin film via modification of the process parameters. Xia et al. observed a change in the growth mechanism from Frank‐van‐der‐Merwe to Volmer‐Weber growth when lowering the substrate temperature from 600 to 300 °C, which resulted in 2D and 3D thin films, respectively.^[^ ^273^ ^]^ Full batteries with a LiPON electrolyte and a Li anode showed improved cycling behavior due to the 3D structure. Deep penetration of the LiPON layer into the 3D cathode significantly increased the surface area. The capacity retention after 500 cycles at 1 C was 73% and 90% for the 2D and 3D structure, respectively. The 3D structure was also applied on an Pt‐coated stainless steel substrate, showing a constant discharge capacity at different stages of bending.^[^ ^273^ ^]^

PVD processes require expensive equipment. Screen printing and ink jet printing are investigated as cost effective methods for electrode fabrication in particular. For example, cathodes with LFP employed as the active material were produced at the laboratory scale by both methods.^[^ ^274^ ^]^

### 5.2 Bulk Batteries

To provide an overview of processing issues in the development ceramic batteries, a brief summary regarding the progress in bulk battery cell development to date is discussed in this section. For further reading, a recently published detailed review by Chen et al. is recommended.^[^ ^275^ ^]^

Only a few full ceramic batteries based on NaSICON electrolytes were described in the literature. For this class of electrolyte materials, the high reactivity with Li‐metal anodes is the main challenge. In most studies on NaSICON materials, model cells with a protective layer made from a type of polymer^[^ ^208^ ^]^ or LiPON^[^ ^213^ ^]^ at the anode‐electrolyte interface were investigated. The discharge capacities of these cells were often lower than the theoretically achievable values, which is most likely due to processing‐induced interface resistance effects.^[^ ^207^, ^208^, ^213^ ^]^ To prevent detrimental reactions at the interface, full batteries were assembled without high‐temperature annealing on the cathode side. For example, the so‐called “all‐phosphate battery” consisted of Li_3_V_2_(PO_4_)_3_ and LiTi_2_(PO_4_)_3_ electrodes, which were densified by pressing and not subjected to a high‐temperature step.^[^ ^276^ ^]^ The cell delivered 63.5 mAh g^−1^ (46% of the theoretical capacity) after 500 cycles.^[^ ^276^ ^]^ The deposition of the cathode material by aerosol deposition was also explored. A full cell consisting of a Nb‐coated NMC111 cathode, a NaSICON electrolyte sheet and a LiPON‐protected Li‐metal anode showed discharge capacities of 87 mAh g^−1^ at 25 °C and 138 mAh g^−1^ at 60 °C at a discharge rate of 0.025 C.^[^ ^277^ ^]^ At the latter temperature 20 charge/discharge cycles were demonstrated.

Even though perovskite electrolytes have been investigated for about 30 years, only limited progress has been made toward realizing full cell assemblies so far. Kotobuki et al. infiltrated honeycomb structured LLT with LMO by employing a low‐temperature sol–gel process. However, only a small amount of the theoretical capacity was utilized with such cells.^[^ ^278^ ^]^ Low capacities were also observed for all‐solid‐state batteries with a thin film LMO cathode and a thin film SnO_2_ anode.^[^ ^279^ ^]^ Details regarding the processing of the electrodes were not described, and therefore one can only speculate as to what the causes of the low capacity might be.

Substantial progress was made with garnet‐based batteries due to the high electrolyte stability toward Li‐metal and relatively high thermal stability during co‐processing with oxidic cathode materials. The first reported all‐solid‐state cells were produced based on a garnet‐electrolyte pellet, which was coated with a positive electrode material.^[^ ^218^, ^280^ ^]^ The cells demonstrated the applicability of garnet electrolytes in all‐solid‐state batteries. However, higher electrode loadings (i.e., the mass of cathode material per area with respect to the solid electrolyte) have to be realized for practical applications. The application of a composite cathode, consisting of a solid electrolyte and CAM, is a rife solution. High electrode loadings of up to 12.6 mg cm^−2^ were realized by rapid processing of the cathode at elevated temperatures.^[^ ^221^ ^]^ High capacities of up to 1.63 mAh cm^−2^ were demonstrated at 50 °C at low discharge rates (50 µA cm^−2^). However, these cells suffered from strong degradation within the first 100 cycles, which was possibly caused by mechanical failure.^[^ ^221^ ^]^ To decrease the processing temperature of composite cathodes, the utilization of sintering additives,^[^ ^281^ ^]^ the infiltration of CAM into porous structures^[^ ^282^ ^]^ and aerosol deposition^[^ ^283^ ^]^ were employed. The battery performance data obtained from these studies clearly demonstrate that these approaches enable significant progress toward realizing practically viable solid‐state batteries. However, there is still room for improvement in terms of cathode utilization and increasing the electrode loading.

## 6 Conclusions

In this review, we emphasized the role of various PVD techniques in the context of improving the fundamental understanding of various processes in ceramic solid‐state batteries. PVD enables a wide range of applications in battery development, from describing fundamental properties of single materials to high‐throughput material development and the fabrication of model cells.

In order to further advance the development of solid‐state batteries, we propose the following recommendations for application of PVD processes:

‐ Material screening by high‐throughput processing, also in combination with computational methods, for example, data mining, should be used more frequently.
‐ The deposition processes for intercalation electrodes that require a crystallization step have to be optimized regarding the crystallization temperature. Only if these processes are available, trustworthy conclusions from model cells about the electrochemical properties of the interface can be made.
‐ The development of diffusion barriers at the interface between the CAM and the electrolyte is crucial to enable processing of these materials at high sintering temperatures. The available publications discussing these layers are rarely considering chemical and electrochemical analysis of the resulting interfaces. Further, interlayers that suppress detrimental diffusion effects at the required sintering temperature have not yet been identified. All of these issues should be investigated in greater depth.

Beyond the discussion of single components and interfaces we also presented the progress on the device scale. State‐of‐the‐art solid‐state batteries, both academic and commercial types, were assessed in view of energy and power density as well as long‐term stability. Finally, recent efforts to improve the power and energy density through the development of 3D‐structured cells and the investigation of bulk cells were discussed. The development of LiPON was an important step in battery technology, not only because it made the commercialization of thin film all‐solid‐state batteries possible, but also because of the unique possibility to monitor the properties of active materials at high temperatures, high voltages, and under mechanical stress. While LiPON based cells are preferred for applications with relatively low energy consumption, ceramic electrolytes are regarded as promising candidates for batteries with high energy density for disruptive technologies in the future. In this context, the application of PVD technology provides key contributions, from material development to the fabrication of complete batteries.

## Conflict of Interest

The authors declare no conflict of interest.
